# Opponent control of behavioral reinforcement by inhibitory and excitatory projections from the ventral pallidum

**DOI:** 10.1038/s41467-018-03125-y

**Published:** 2018-02-27

**Authors:** Lauren Faget, Vivien Zell, Elizabeth Souter, Adam McPherson, Reed Ressler, Navarre Gutierrez-Reed, Ji Hoon Yoo, Davide Dulcis, Thomas S. Hnasko

**Affiliations:** 1 Department of Neurosciences, University of California, La Jolla, San Diego, CA 92093 USA; 2 Department of Psychiatry, University of California, La Jolla, San Diego, CA 92093 USA; 3 Research Service VA San Diego Healthcare System, La Jolla, San Diego, CA 92161 USA

## Abstract

The ventral pallidum (VP) lies at the interface between sensory, motor, and cognitive processing—with a particular role in mounting behavioral responses to rewards. Though the VP is predominantly GABAergic, glutamate neurons were recently identified, though their relative abundances and respective roles are unknown. Here, we show that VP glutamate neurons are concentrated in the rostral ventromedial VP and project to qualitatively similar targets as do VP GABA neurons. At the functional level, we used optogenetics to show that activity in VP GABA neurons can drive positive reinforcement, particularly through projections to the ventral tegmental area (VTA). On the other hand, activation of VP glutamate neurons leads to behavioral avoidance, particularly through projections to the lateral habenula. These findings highlight cell-type and projection-target specific roles for VP neurons in behavioral reinforcement, dysregulation of which could contribute to the emergence of negative symptoms associated with drug addiction and other neuropsychiatric disease.

## Abstract (web summary)

The ventral palladium (VP) is involved in reward and contains mostly GABAergic neurons, but also glutamatergic neurons. Here, the authors characterize glutamatergic VP neurons and show that glutamatergic versus GABAergic projections play roles in avoidance and reinforcement, respectively.

## Introduction

The ventral pallidum (VP) is a heterogeneous region of the basal forebrain heavily innervated by GABAergic Substance P (SP)-expressing fibers from the nucleus accumbens (NAc). The VP integrates limbic and cognitive signals, anatomically rests at the interface between motivation and action, and is thus centrally positioned in the limbic system to control motivated behaviors^1–8^. Indeed, the VP has been shown capable of potently modulating reward-seeking behaviors. For example, rats will work to electrically self-stimulate the VP^9^, pharmacological activation or disinhibition can trigger feeding in sated animals^10–12^, and microinjection of opioids amplify hedonic “liking” reactions in rats^12,13^. Conversely, VP lesions reduce cocaine or heroin self-administration^14^, and can cause aphagia and aversive reactions to sucrose^15^. Further, neural activity in the VP is sensitive to hedonic stimuli and reward-predictive cues^12,16–21^.

These studies demonstrate that VP neurons play a critical role in reward. However, the VP is a heterogeneous structure containing a multitude of neurochemical phenotypes and functional zones. Earlier studies identified the medial (VPm) and lateral portions (VPl) as distinct sub-territories, receiving projections from the NAc shell and core, respectively, and with some distinct projection targets^22,23^. Also, while the entire VP is pervaded by SP-expressing fibers, the ventromedial portion (VPvm) is strongly innervated by neurotensin-positive fibers and the dorso-lateral portion (VPdl) by calbindin-positive fibers^3^. At the functional level, VPvm and VPdl neurons show distinctive response characteristics during cocaine self-administration^24^. Electrophysiological characteristics also differ depending on their rostro-caudal localization^25,26^, and pharmacological manipulation of the rostral (RVP) or caudal (CVP) VP induce different behavioral responses. For example, SP microinjection in the caudal but not rostral VP elicited conditioned place preference (CPP)^27^. Further, mu opioid receptor agonists increase reward and consummatory behavior in the CVP, but suppress “liking” reactions and eating in the RVP^12,28^. Another study demonstrated that chemogenetic inhibition of the RVP blunts cue-induced reinstatement, and inhibiting the CVP blocks cocaine-primed reinstatement^29^.

Another source of heterogeneity that has received less attention is the neurochemical identity of VP cells. The VP is composed principally of GABAergic projection neurons, but cholinergic neurons are also present as are numerous peptidergic markers^2^. In addition, the vesicular glutamate transporter VGLUT2-expressing glutamate neurons were identified in the VP^30^. Interestingly, while ventral tegmental area (VTA)-projecting VP neurons have been identified as functionally GABAergic^31^, VP glutamate projections to VTA were also reported^32^. But it remains unknown whether VP glutamate neurons functionally cooperate with or oppose VP GABA projections in reward processes.

In this study, we first sought to better define the anatomical location, relative proportion, and molecular phenotype of excitatory VGLUT2^+^ VP cells. Then using cell type-specific viral approaches, we compared the projection targets and synaptic connectivity of these neurons in relation to GABAergic and cholinergic neurons in the VP. Finally, we optogenetically manipulated VP glutamate versus GABA cells, or specific VP projections, to demonstrate that these cell types can play functionally opponent roles in the control of motivated behavior.

## Results

### Glutamate neurons are concentrated in the ventromedial RVP

**Fig. 1:**
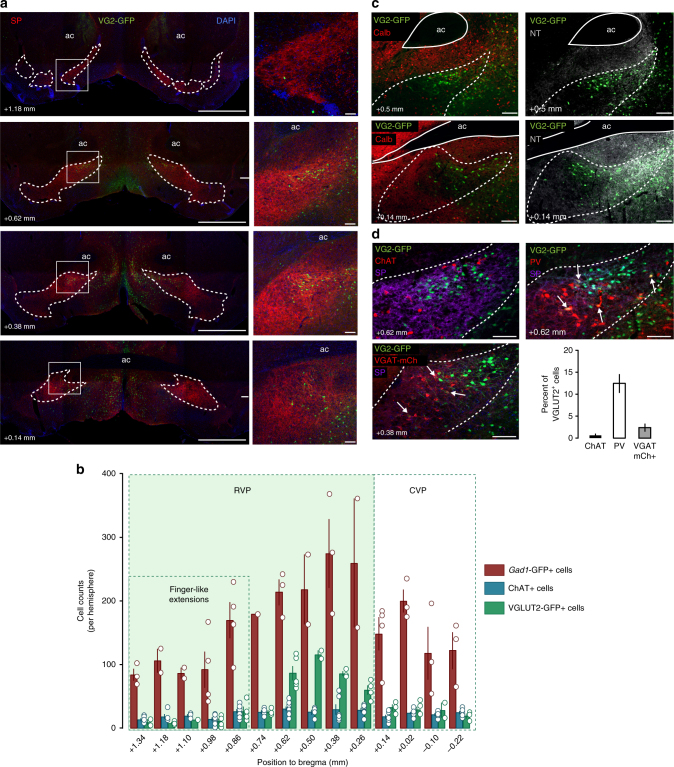
Localization and histochemical characterization of VP glutamate neurons. **a** Coronal images through the rostro-caudal axis of the VP of VGLUT2-GFP (VG2-GFP; green) reporter mice. **b** VGLUT2^+^ neurons were observed throughout the rostro-caudal axis but most abundant in centro-rostral VP (1791 VGLUT2+ cells, 44 sections, five mice). *Gad1*-GFP mice were used to assess GABA neurons (6196 *Gad1*-GFP+ cells, 39 sections, four mice) and all sections were stained and assessed for the cholinergic marker ChAT (1901 ChAT+ cells, 83 sections,nine mice). **c** Identification of the dorso-lateral subregion of the VP (VPdl) using antibodies directed against Calbindin (Calb), and the ventromedial subregion (VPvm) using antibodies directed against neurotensin (NT) show the densest cluster of VGLUT2^+^ cells in the VPvm. **d** Co-localization of VGLUT2+ cells with ChAT (1078 VGLUT2+ cells, eight VGLUT2+ ChAT+ cells, 36 sections, three mice), Parvalbumin (PV) (1692 VGLUT2+ cells, 200 VGLUT2+ PV+ cells, 44 sections, four mice), or VGAT (820 VGLUT2+ cells, 19 VGLUT2+ VGAT mCh+ cells, 16 sections, three mice). Substance P (SP) was used to delimitate VP boundaries in **a** and **d**, DAPI nuclear stain is shown in **a**, all distances are relative to Bregma, and cell counts or percent of VGLUT2^+^ cells expressed as mean ± SEM. Scale = 1 mm (**a**; left panels) and 100 µm (**a**; right panels, **c** and **d**). See also Supplementary Figure 1

The VP is considered a predominantly GABAergic structure and though several reports have suggested that it also contains glutamate-releasing neurons^2,30,32^, the proportions of each have not been determined. To do so, we used a bacterial artificial chromosome (BAC) transgenic mouse line expressing enhanced green fluorescent protein (GFP) under the control of *Slc17a6* (VGLUT2) regulatory elements, which we first validated by dual in situ hybridization (Supplementary Fig. 1A, B). We then used immunohistochemistry and observed VGLUT2-GFP-expressing cells throughout the rostro-caudal axis of the VP, as delineated by SP (Fig. 1a). To quantify the relative abundance of neurotransmitter-defined VP cell types we used both VGLUT2-GFP and *Gad1*-GFP (glutamatic acid decarboxylase) reporter mice, immunolabeling against GFP, the cholinergic marker choline acetyltransferase (ChAT), the neuronal marker NeuN and SP (Fig. 1b, Supplementary Fig. 1C). In sum, we found that VGLUT2^+^ glutamate neurons represent 15 ± 2%, *Gad1*^+^ cells 74 ± 1%, and ChAT^+^ cells 11 ± 1% of neurotransmitter-defined cells. However, their relative proportions varied widely by Bregma point with glutamate neurons most abundant in the central RVP (Fig. 1b), a result we again validated using dual in situ hybridization (Supplementary Fig. 1D). Using calbindin as a marker for the dorso-lateral VP (VPdl) and neurotensin to label ventromedial VP (VPvm)^3^, the densest VGLUT2^+^ cell cluster localized to the VPvm (Fig. 1c).

To further characterize the neurochemical properties of these VGLUT2^+^ cells, we tested for their co-localization with ChAT or the calcium-binding protein parvalbumin (PV). A very small proportion of VGLUT2^+^ cells co-labeled for ChAT (0.7 ± 0.4%), while a substantial minority population co-labeled for PV (12.5 ± 2.1%) (Fig. 1d, arrows). To test for co-localization of glutamate and GABA markers, we crossed VGLUT2-GFP mice with mice expressing Cre recombinase under the control of *Slc32a1* regulatory elements (vesicular GABA transporter; VGAT-Cre) and expressed a fluorescent tag in the VP using a Cre-dependent adeno-associated virus (AAV). We found relatively low rates of co-expression with only 2.4 ± 0.9% of VGLUT2^+^ cells co-labeled for the GABA marker. Labeling by dual in situ hybridization was also consistent with low rates of VGLUT2/VGAT co-localization in the VP (Supplementary Fig. 1A, B). These findings suggest that VGLUT2^+^ VP neurons are essentially distinct from cholinergic cells, that a small fraction co-localize for GABA markers, and that PV is expressed in subpopulations of both inhibitory and excitatory VP neurons, consistent with recent studies^33,34^.

### VP glutamate and GABA neurons share similar projection sites

**Fig. 2:**
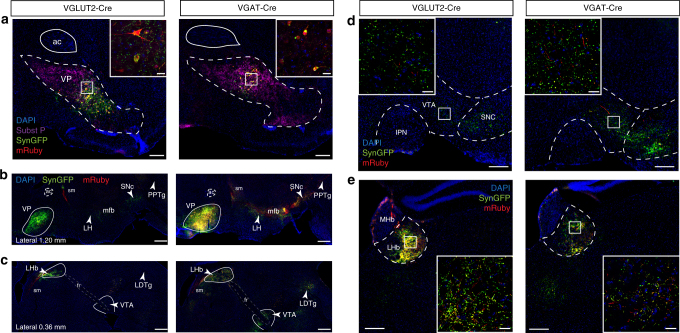
Projections of VP glutamate and GABA neurons. **a** Coronal section showing expression of cytosolic mRuby and Synaptophysin:GFP in VP glutamate and GABA neurons using VGLUT2-Cre or VGAT-Cre mouse lines. **b**–**c** Sagittal sections showing VP glutamate and GABA neurons project to similar targets throughout the brain including lateral hypothalamus (LH), ventral tegmental area (VTA), substantia nigra pars compacta (SNc), lateral habenula (LHb), pedunculopontine nucleus (PPTg), and laterodorsal tegmental nucleus (LDTg) as seen in sagittal sections from two different lateral coordinates. The anterior commissure (ac) and fasciculus retroflexus (fr)—habenulointerpeduncular tract—are highlighted with dashed lines. Approximate borders of the VP, LHb, and VTA are delineated with plain white lines. Coronal sections through two key VP afferents in the **d**. VTA and **e**. LHb. IPN interpeduncular nucleus, MHb medial habenula, ac anterior commissure, sm stria medullaris, mfb medial forebrain bundle, fr fasciculus retroflexus. Scale = 200 µm (widefield), and 20 µm (insets). See also Supplementary Figures 2, 3, and 4

We next sought to compare the projection targets of neurotransmitter-defined VP cell types. Using a Cre-dependent viral vector, we expressed cytosolic mRuby and the synaptic marker synaptophysin:GFP in the VP of mice expressing Cre recombinase under the control of VGLUT2 or VGAT regulatory elements. This approach enabled us to distinguish putative release sites (synaptophysin:GFP^+^) from passing fibers (mRuby^+^) of defined VP cell types. Expression in either VP GABA or glutamate neurons led to a broadly similar pattern of labeling and synaptophysin:GFP puncta were identified in diverse regions, including mPFC, lateral habenula (LHb), medial amygdala (MeA), lateral hypothalamus (LH), substantia nigra pars compacta (SNc), VTA, pedunculopontine nucleus (PPTg), and latero-dorsal tegmental nucleus (LDTg) (Fig. 2 and Supplementary Fig. 2). A very similar pattern of projections was observed when ChR2:YFP was expressed in VGLUT2-Cre or VGAT-Cre neurons. ChAT-Cre neurons in the VP displayed a notably distinct and sparser pattern of projections, primarily targeting the basolateral amygdala and prefrontal cortex (Supplementary Fig. 3 and Supplementary Table 1), consistent with recent studies targeting cholinergic neurons in the basal forebrain^35^. Interestingly, we also detected abundant synaptophysin:GFP locally in the VP (Fig. 2a, b), suggestive of local connectivity. This conclusion is supported by the existence of both intra-VP IPSCs and EPSCs following photostimulation of VGAT-Cre or VGLUT2-Cre VP neurons (Supplementary Fig. 4). These studies extend previous works^4,35^ and demonstrate that VGAT^+^ and VGLUT2^+^ VP neurons share similar projection targets, thus suggesting heterogeneous inhibitory–excitatory control from the VP to numerous brain regions critical for reward-related behaviors.

### VP GABA and glutamate neurons drive opposite behaviors

To directly explore the role of VP glutamate and GABA populations in behavioral reinforcement, we compared the effect of their activation or inhibition using optogenetics in real-time place preference (RTPP) and intracranial self-stimulation (ICSS) tasks. Activation of the VP has been shown reinforcing by electrical stimulation and substantial subsets of VP neurons transiently burst at rates between 5 and 50 Hz in response to rewards and reward-predictive cues^9,16,19,21,36,37^. Based on their projection patterns and greater abundance, we hypothesized that stimulation of VP GABA neurons would promote reward seeking, while stimulation of VP glutamate neurons may oppose this. Alternatively, both cell types might support reward behaviors.

**Fig. 3:**
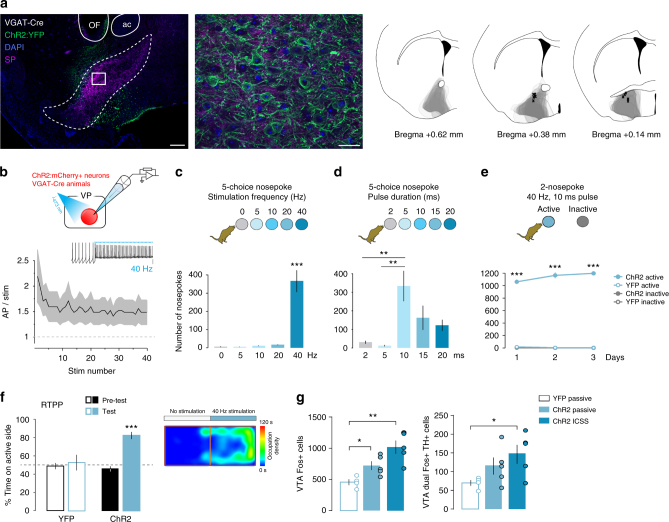
Stimulation of VP GABA neurons elicits positive reinforcement. **a** Expression of ChR2:YFP in the VP of VGAT-Cre mouse imaged under epifluorescent (left, scale 200 µm) or apotome (right, scale 20 µm) illumination. Counterstaining with SP (purple) and DAPI (blue), ac anterior commissure, OF optic fiber track. Spread of ChR2:YFP expression in the VP of VGAT-Cre animals (overlapped gray areas for each animal, right panel) and optic fiber tip placements (*x* marks the spot). **b** Firing of ChR2:mCh^+^ VP GABA neurons in response to 40-Hz photostimulation (*n* = 9 cells); SEM represented in gray. Inset shows representative trace; scale = 20 mV. **c** In a five-choice nosepoke ICSS task for VP GABA neuron stimulation mice prefer 40-Hz stimulation (*n* = 7). **d** When choosing between pulse durations, mice preferred 10 ms pulse width (*n* = 7). **e** Over 3 daily 1-h two-nosepoke ICSS sessions, mice made 1141 ± 41 nosepokes for stimulation of VP GABA neurons (*n* = 6 YFP controls and eight ChR2). **f** In an RTPP assay, mice preferred the compartment paired with stimulation of VP GABA neurons (*n* = 9 YFP controls and 10 ChR2). Right panel shows occupation density of an example mouse during the test trial. **g** Fos^+^ cells in the VTA increased following ICSS or passive activation of VP GABA neurons (*n* = 4 VGAT YFP controls, *n* = 5 VGAT ChR2 passive and ICSS each). The number of VTA cells double positive for the dopamine marker TH and Fos increased following ICSS for VP GABA neuron activation. *p < 0.05, **p < 0.01, ***p < 0.001. See also Supplementary Figures 5 and 6

To first validate our ability to evoke cell type-specific VP activation with optogenetics, we expressed ChR2 in VGAT^+^ VP neurons (Fig. 3a) and made acute slice preparations through the VP for ex vivo electrophysiological recordings. When recording from ChR2:YFP-expressing VP neurons, we found that they readily fired in response to light trains at frequencies up to 40 Hz (Fig. 3b). We then tested animals for ICSS using a five-choice instrumental nosepoke assay that allows for the simultaneous comparison across multiple parameters of light delivery. Given the choice between different frequencies of stimulation, mice preferred the nosepoke hole coupled to the highest frequency available, for example, 40 Hz (repeated measure (RM) one-way ANOVA, *F*_(4, 24)_ = 33.2, *p* < 0.0001; Fig. 3c) or 20 Hz (Supplementary Fig. 5A). (Note: Additional statistical detail is presented in Supplementary Table 2). When frequency was kept constant (40 Hz, 1 s) and pulse width varied, mice preferred the nosepoke coupled to the 10-ms pulse width (RM one-way ANOVA, *F*_(4,24)_ = 5.6, *p* = 0.0025; Fig. 3d). Using these optimized parameters in a two-nosepoke ICSS task (Fig. 3e), with active and inactive nosepoke holes, ChR2-expressing mice showed intensive ICSS, making considerably more nosepokes on the active hole compared to YFP-expressing controls (RM three-way ANOVA, viral treatment × nspk type, *F*_(1,24)_ = 1592, *p* < 0.0001). Consistent with these findings, mice showed a strong preference for the laser-stimulated side during an RTPP task (RM two-way ANOVA, viral treatment × day, *F*_(1,17)_ = 14.2, *p* = 0.0015; Fig. 3f), where movement into or out of the laser-paired compartment turned on or off the optogenetic stimulation of VP GABA neurons, respectively. In a separate cohort of animals, we varied frequency and light intensity and observed that, among the parameters tested, 40-Hz at 10 mW induced the greatest side preference (Supplementary Fig. 5B). Further, following 3 days of conditioning in a CPP assay (i.e., with mice confined to chambers during conditioning) using the previously noted stimulation parameters, mice developed a conditioned place preference for the compartment associated with stimulation (RM two-way ANOVA, viral treatment × day, *F*_(1, 13)_ = 6.2, *p* = 0.03) (Supplementary Fig. 5C).

The reinforcing properties associated with VP GABA neuron stimulation were associated with an increased activity of VTA neurons. Both passive (1 s 40 Hz 10 ms pulse width stim every 5 s; 360 stim/30 min) and active (ICSS task; 379 ± 63 stim/30 min) stimulation of VGAT^+^ VP neurons produced an increase in the number of Fos^+^ cells in the VTA (unpaired *t*-test, YFP vs. ChR2 passive, *t*_(7)_ = 2.9, *p* = 0.02; YFP vs. ChR2 ICSS, *t*_(7)_ = 4.4, *p* = 0.003), and both TH^+^ and TH^−^ neurons responded (unpaired *t-*test, YFP vs. ChR2 ICSS, *t*_(7)_ = 2.6, *p* = 0.035) (Fig. 3g). These data are consistent with prior studies showing that stimulation of the VP induced DA release^38^ and, together with the profound behavioral reinforcement observed, suggest a polysynaptic disinhibition mechanism is involved. We also observed substantial Fos induction in the LHb following either active ICSS or passive stimulation of VGAT^+^ VP cell bodies (Supplementary Fig 6A). This was surprising because activation of the LHb is sufficient to produce avoidance behavior, but these animals were receiving a reinforcing stimulus and engaged in an appetitive self-stimulation task. Importantly, however, when we directly stimulated these GABAergic terminals in LHb we evoked only minimal Fos induction that was not statistically significant (Supplementary Fig. 6B–D), suggesting that the LHb Fos induced by VP GABA neuron activation was also polysynaptic.

**Fig. 4:**
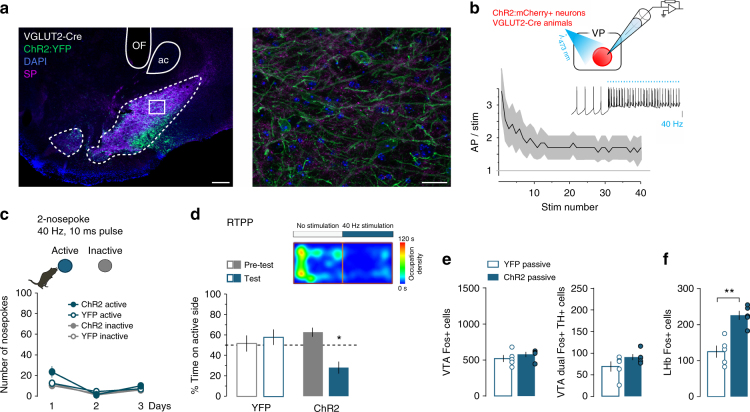
Stimulation of VP glutamate neurons induces place avoidance. **a** Expression of ChR2:YFP in the VP of VGLUT2-Cre mouse imaged under widefield (left, scale 200 µm) or apotome (right, scale 20 µm) illumination. **b** Action potential discharge of ChR2:mCherry^+^ VP glutamate neuron in response to 40-Hz photostimulation (*n* = 7 cells); SEM represented in gray. Inset shows representative trace; scale = 20 mV. **c** Over three daily 1-h two-nosepoke ICSS sessions, mice did not demonstrate robust self-stimulation for VP glutamate neurons (*n* = 5 YFP controls, *n* = 6 ChR2). **d** VGLUT2-Cre mice avoided the compartment paired with photostimulation of VP glutamate neurons in the RTPP assay (*n* = 5 YFP and ChR2 animals). Right panel shows occupation density of an example mouse during a test trial. **e** Passive activation of VP glutamate neurons failed to increase Fos^+^ cell counts in the VTA (*n* = 5). **f** However, the number of LHb cells positive for Fos increased following passive stimulation of VP glutamate neurons. *p < 0.05, **p < 0.01, ***p < 0.001. See also Supplementary Figure 5

We next tested the effects of ChR2-mediated photostimulation on VP glutamate neurons (Fig. 4a). As with the GABA neurons, ChR2-expressing VGLUT2^+^ VP neurons were able to maintain firing rates at or above 40 Hz in response to 40-Hz photostimulus trains in slice preparation (Fig. 4b). When tested in the two-nosepoke ICSS task, mice failed to self-stimulate VP glutamate neurons (40 Hz, 10 ms pulse width) (Fig. 4c). However, when tested by RTPP, mice displayed a marked avoidance for the chamber associated with photostimulation (RM two-way ANOVA, viral treatment × day, *F*_(1,8)_ = 13.8, *p* = 0.006) (Fig. 4d). In a separate cohort of animals, different frequencies and light intensities were tested and, among the parameters tested, 40 Hz 10 ms pulse width at 10 mW induced the strongest avoidance, and no preference was observed under any condition (Supplementary Fig. 5D). To test how activation of VP glutamate neurons altered activity in postsynaptic regions we tested Fos induction in the VTA and LHb following the passive stimulation of VGLUT2 VP neurons (1 s 40 Hz 10 ms pulse width delivered every 5 s for 30 min). In contrast to VP GABA neuron stimulation, activation of VP glutamate neurons did not elicit an increase in the number of Fos-labeled TH^+^ or TH^−^ VTA cells (Fig. 4e). However, stimulation of VGLUT2^+^ VP cells did produce a significant increase in the number of Fos^+^ cells in the LHb (Unpaired *t*-test, *t*_(8)_ = 5.2, *p* = 0.0008; Fig. 4f).

**Fig. 5:**
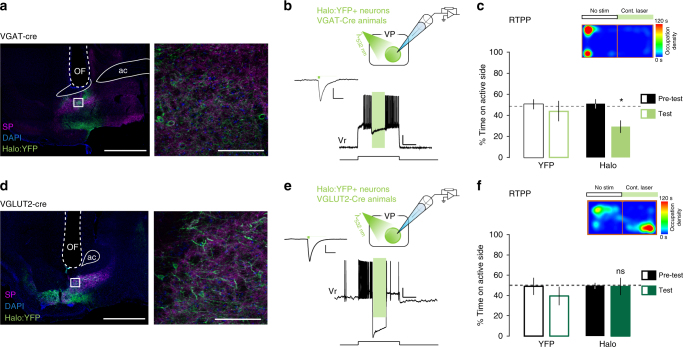
Inhibition of VP GABA but not glutamate neurons induce place avoidance. **a** Expression of Halo:YFP (green) in VP GABA neurons. Counterstaining with SP (purple) and DAPI (blue); OF optic fiber track; ac anterior commissure. **b** 1-s light pulse inhibits firing of VP GABA neuron; scale = 20 mV, 1 s. Inset shows photocurrent from Halo:YFP-expressing VP GABA neuron in response to 5-ms pulse of green light; scale = 4 pA, 50 ms. **c** Mice avoid the compartment paired with bilateral inhibition of VP GABA neurons in the RTPP (*n* = 5 YFP, *n* = 6 Halo). **d** Expression of Halo:YFP (green) in VP glutamate neurons. **e** 1-s light pulse inhibits firing of VP glutamate neuron; scale = 20 mV, 1 s. Inset shows hyperpolarizing photocurrent from Halo:YFP-expressing VP glutamate neuron in response to 5-ms pulse of light; scale = 20 pA, 50 ms. **f** Bilateral inhibition of VGLUT2^+^ VP cells did not induce preference or avoidance in the RTPP (*n* = 5 YFP, *n* = 8 Halo). Image scale bars = 500 µm (left panels) and 100 µm (right panels); *p < 0.05

To test how spontaneous or tonic activity in neurotransmitter-defined VP neurons may contribute to the affective state of the animal, we tested the effect of inhibiting VP cell types in mice expressing Halorhodopsin (eNpHR3.0:YFP; Halo) in either VGAT^+^ (Fig. 5a–c) or VGLUT2^+^ VP neurons (Fig. 5d–f). Whereas ChR2-driven activation of VP GABA neurons led to place preference, the inhibition of VGAT^+^ VP neurons-induced avoidance in the RTPP assay (RM two-way ANOVA, day × viral treatment, *F*_(1,9)_ = 9.4, *p* = 0.01) (Fig. 5c). However, despite our previous observation that activation of VGLUT2^+^ VP neurons led to place avoidance, neither RTPP nor avoidance resulted from their inhibition (Fig. 5f, RM two-way ANOVA, day × viral treatment, *F*_(1,11)_ = 0.86, *p* = 0.37, NS), suggesting that VP glutamate neurons may be inactive or that their tonic activity does not convey a valence signal under assay conditions. Altogether these data demonstrate that VP GABA neurons can potently drive behavioral reinforcement, and that activation of VP glutamate neurons may functionally oppose VP GABA neurons and play a role in aversive signal processing.

### VTA or LHb terminal activation drive differential behaviors

We showed that both GABA and glutamate neurons in the VP project to multiple brain areas whose activity can regulate motivation and reward (Fig. 2, Supplementary Table 1). Two of the densest targets of both VP GABA and glutamate neurons were the VTA and LHb, regions generally associated with positive or negative valence, respectively^39,40^. To better map and compare cell type-specific inputs to these two key brain regions we made use of a DO-DIO viral construct that enabled labeling of Cre-on and Cre-off cells with different fluorophores in the same animal (Supplementary Fig. 7A, B). These data suggest that VP glutamate neurons more heavily and broadly innervate the LHb, while VP GABA neurons more heavily innervate the VTA (Supplementary Fig. 7C, D), consistent with their differential roles in the regulation of aversion and reward. The VP inputs to these structures likely represent largely separate populations of cells as the injection of different color retrobeads into the LHb and VTA led to only very low levels of co-localization in VP (Supplementary Fig. 7E). Further, the injection of retroAAV-Cre into VTA and AAV-DIO-ChR2:mCherry into VP led to dense fiber labeling in VTA but few detectable fibers in LHb (Supplementary Fig. 7F).

**Fig. 6:**
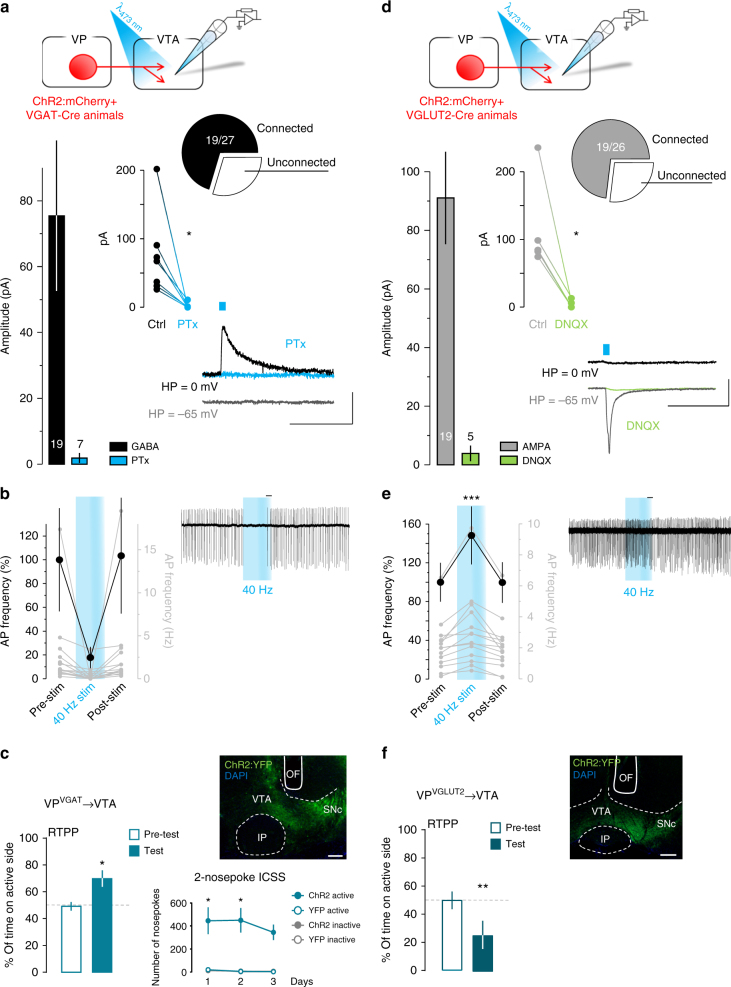
Activation of VP projections to VTA recapitulates behavioral responses. **a** Whole-cell recordings in the VTA reveal that single-pulse (5-ms, blue dash) photostimulation of VGAT^+^ VP terminals triggered PTx-sensitive IPSCs. Pie chart show the fraction of responding neurons, bar graphs show peak IPSC amplitude, insets shows individual cells pre- and post- PTx (*n* = 7, paired *t-*test, *t*_(6)_ = 3.2, *p* = 0.019); and representative trace; numbers inside bars represent sample size. **b** Cell-attached recordings in the VTA show that photostimulation of GABA terminals from the VP consistently reduced action potential firing frequency in the VTA (*n* = 14, Wilcoxon matched-pairs signed-rank test, *p* = 0.0001). The black scatter plot shows mean change in firing rate 5-s before, during, and after 40-Hz photostimulation, gray axis and plots show individual neuron responses, inset (right) shows a representative trace. **c** VGAT-Cre mice displayed a preference for the compartment paired with photostimulation of VP terminals in the VTA on the RTPP assay (*n* = 11) and showed ICSS over three daily sessions (*n* = 4 YFP, *n* = 8 ChR2). **d** Whole-cell recordings, as described in **a**, show that photostimulation of VGLUT2^+^ VP terminals in the VTA triggered DNQX-sensitive EPSCs (*n* = 5, paired *t*-test, *t*_(4)_ = 3.75, *p* = 0.019); **e** and cell-attached recordings, as described in **b**, show that train of photostimulation increased action potential firing frequency in the VTA (*n* = 14, Wilcoxon matched-pairs signed-rank test, *p* = 0.0001). **f** VGLUT2-Cre mice showed avoidance for the compartment paired with photostimulation of VP terminals in the VTA on the RTPP assay (*n* = 6). Example images of ChR2:YFP and optic fiber (OF) tracks in the VTA of **c** VGAT-Cre or **f** VGLUT-Cre mice; DAPI (blue), scale = 200 µm. Scales for **a** and **d** are 50 pA, 50 ms and 200 pA, 50 ms, respectively; scale for **b** and **e** is 1 s; *p < 0.05, **p < 0.01, ***p < 0.001. See also Supplementary Figure 7 and Supplementary Table 3

We next assessed the postsynaptic effects of stimulating ChR2-expressing VGAT^+^ and VGLUT2^+^ VP inputs on VTA neurons using slice electrophysiology. Single-pulse photostimulation of VGAT^+^ terminals led to IPSCs in 70% of neurons recorded in VTA, subsets of which were tested pharmacologically and sensitive to Picrotoxin (Fig. 6a; additional postsynaptic properties in Supplementary Table 3). In another set of experiments, we used cell-attached recordings to test whether the synaptic effects of 40-Hz train photostimulation of VGAT^+^ VP terminals could alter spontaneous firing of the postsynaptic cells. Indeed, we found a reversible inhibition of spontaneous firing during photostimulation of VGAT^+^ terminals in the VTA (Fig. 6b). In vivo, we found that similar to optogenetic stimulation of VP cell bodies, activation of VGAT^+^ VP terminals in the VTA led to preference in the RTPP (Fig. 6c) (paired *t-*test, *t*_(10)_ = 3.1, *p* = 0.01), and to robust ICSS on the two-nosepoke assay compared to controls (RM three-way ANOVA, viral treatment × nspk type, *F*_(1,20)_ = 11.8, *p* = 0.003).

We used the same approaches to examine the VGLUT2^+^ projections to VTA from VP. Ex vivo photostimulation of VGLUT2^+^ VP inputs to VTA (Fig. 6d) revealed EPSCs in 73% of postsynaptic cells, a connectivity rate similar to that detected for VP GABA inputs. Corresponding to this observation, cell-attached recordings showed that VTA neurons consistently increased firing in response to train stimulation of VGLUT2^+^ VP terminals (Fig. 6e). In vivo, the behavioral results of stimulating VGLUT2^+^ projections to VTA were consistent with our observations when targeting VP soma; activation of VGLUT2^+^ VP terminals in the VTA (Fig. 6f) also induced behavioral avoidance on the RTPP assay (paired *t*-test, *t*_(5)_ = 4.1, *p* = 0.009). Since VGLUT2^+^ cell body stimulation did not support ICSS, and VTA terminals led to avoidance in RTPP, ICSS was not assessed in this cohort.

**Fig. 7:**
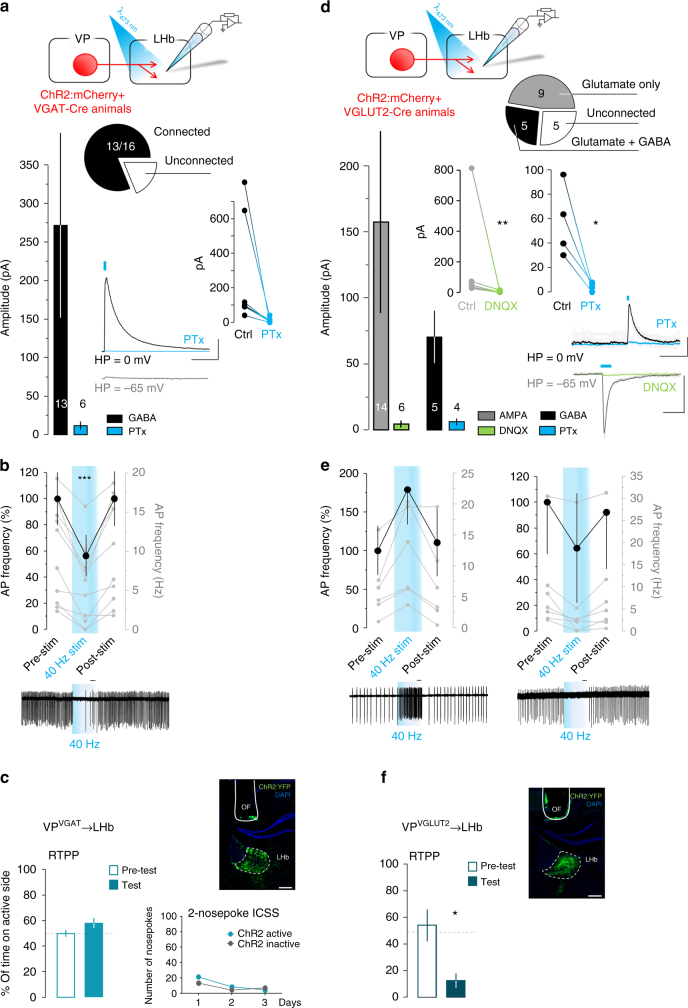
Only activation of VP glutamate but not GABA projections to LHb recapitulates behavioral responses. **a** Whole-cell recordings in the LHb reveal that single-pulse (5-ms, blue dash) photostimulation of VGAT^+^ VP terminals triggered PTx-sensitive IPSCs (*n* = 7, paired *t*-test, *t*_(6)_ = 2.19, *p* = 0.07). Pie chart shows the fraction of responding neurons, bar graphs show peak IPSC amplitude, insets show individual cells pre- and post-PTx and representative trace; numbers inside bars represent sample size. **b** Cell-attached recordings in the LHb show that photostimulation of GABA terminals from the VP consistently reduced action potential firing frequency in the LHb (*n* = 12, Wilcoxon matched-pairs signed-rank test, *p* = 0.0005). The bar graph shows mean change in firing rate 5-s before, during, and after 40-Hz photostimulation, gray axis and plots show individual neuron responses, inset (right) shows a representative trace. **c** VGAT-Cre mice did not show a preference for the compartment paired with photostimulation of VP terminals in the LHb on the RTPP assay (*n* = 12) and did not self-stimulate LHb terminals stimulation in the two-nosepoke ICSS task. **d** Whole-cell recordings, as described in **a**, show that photostimulation of VGLUT2^+^ VP terminals in the LHb triggered DNQX-sensitive EPSCs (*n* = 6, paired *t*-test, *t*_(5)_ = 4.73, *p* = 0.005) as well as PTx-sensitive IPSCs (*n* = 4, paired *t*-test, *t*_(3)_ = 3.77, *p* = 0.033); **e** and cell-attached recordings, as described in **b**, show that train of photostimulation increased (*n* = 7) or decreased (*n* = 6) action potential firing frequency in the LHb. **f** VGLUT2-Cre mice showed avoidance for the compartment paired with photostimulation of VP terminals in the LHb on the RTPP assay (*n* = 6). Example images of ChR2:YFP and optic fiber (OF) tracks in the LHb of **c** VGAT-Cre or **f** VGLUT-Cre mice; DAPI (blue), scale = 200 µm. Scales for **a** are 250 pA, 50 ms. Scales for **d** are 200 pA, 50 ms (PTx) and 500 pA, 10 ms (DNQX). Scales for **b** and **e** are 1 s; *p < 0.05, **p < 0.01, ***p < 0.001. See also Supplementary Figure 7 and Supplementary Table 3

We next targeted VP terminals in the LHb for optogenetic stimulation. Single-pulse photostimulation of VGAT^+^ terminals led to IPSCs in 81% of LHb neurons recorded from (Fig. 7a), and cell-attached recordings showed reversible inhibition of spontaneous firing during train photostimulation (Fig. 7b). In vivo we found that, unlike VTA terminal stimulation, optogenetic activation in the LHb did not lead to robust or statistically significant preference in the RTPP (paired *t*-test, *t*_(11)_ = 1.86, *p* = 0.09), nor did it recapitulate the self-stimulation behavior observed in the two-nosepoke ICSS assay (Fig. 7c). Thus while the optogenetic activation of both pathways resulted in significant post-synaptic inhibition, transient activation of the GABAergic projection to VTA appeared to be of greater import in driving motivated behavior. Finally, we used the same approaches to examine the VGLUT2^+^ inputs from VP to LHb. EPSCs were detected in 73% of recorded neurons during ex vivo photostimulation of VGLUT2^+^ VP inputs to LHb (Fig. 7d). Interestingly, though never detected in the VTA, 26% of cells in the LHb showed fast apparent monosynaptic IPSCs (Supplementary Table 3), which were blockable by picrotoxin but not by DNQX. Though cell-attached recordings showed that the net effect of photostimulation was null (7.7 ± 32% increase in firing), LHb neurons could be divided into those that were reversibly excited or inhibited during train stimulation of VGLUT2^+^ VP terminals (Fig. 7e). In vivo, stimulation of VGLUT2^+^ terminals in the LHb induced a strong place avoidance (paired *t*-test, *t*_(5)_ = 5.1, *p* = 0.026) (Fig. 7f), suggesting that the avoidance produced by somatic stimulation of VP glutamate neurons may be mediated, at least in part, through direct activation of LHb.

## Discussion

Multiple studies have shown that VP neurons fire in response to rewards or reward-related stimuli and that VP manipulations can profoundly influence reward-seeking behaviors^2,8^. Because electrical activation of the VP can support ICSS and because the VP is composed principally of GABA projection neurons, we hypothesized that activation of these neurons would provoke appetitive behavior. Indeed, optogenetic activation of VP GABA neurons-induced profound positive reinforcement in CPP, RTPP, and ICSS behavioral assays. Conversely, inhibiting VP GABA cells produced behavioral avoidance, consistent with the hypothesis that VP GABA cells provide tonic inhibitory tone to VTA that serves as a substrate to bidirectionally regulate the value of environmental stimuli^16,17,29,37,41,42^. Stimulation of VP GABA projections to VTA, but not to LHb, recapitulated preference in the RTPP and ICSS tasks, suggesting that the VTA projections most potently drive appetitive behavior. And despite our observations that VP GABA cells provide broad monosynaptic inhibition to the VTA, their in vivo activation increased VTA cellular activity as assessed by Fos labeling. The ability of VTA-projecting VP GABA neurons to drive reinforcement and cellular activation are thus likely mediated via disinhibition, as has been suggested for other inhibitory inputs to VTA^38,43,44^. Indeed, the LH, bed nucleus of the stria terminalis, lateral preoptic area, and VP share similar connection patterns and their cell type-specific manipulation induce similar effects on ICSS and RTPP assays^45–49^. The VP may thus represent one node within an ensemble of lateral hypothalamic and basal forebrain nuclei that serve to orchestrate motivation and appetitive behavior.

The VTA is highly heterogeneous, containing dopamine, GABA, and glutamate projection neurons and intra-VTA connectivity, each of which have been linked to the control of reinforcement^50–52^. On the other hand, the LHb is composed principally of excitatory projection neurons and multiple studies show that LHb activity relates to and drives behaviors linked to aversion, depression, or disappointment^39,40,53^. Depending on the cellular level connectivity, one might predict that the behavioral consequences of activating VP glutamate neurons either functionally oppose or cooperate with VP GABA neurons. Though our slice physiology experiments were not designed to assess molecular phenotypes of the postsynaptic cell, we find that VP GABA and glutamate neurons have high rates of connectivity in both VTA and LHb, consistent with qualitatively broad effects of VP inputs across cell types^54^. At the functional level, we show that activation of glutamate cell bodies in the VP, or their terminals in the VTA or LHb, each induced avoidance, suggesting that VP glutamate neurons work in opposition to VP GABA neurons. However, their inhibition was not sufficient to induce a preference or avoidance, suggesting that VP glutamate cells may be silent under assay conditions or respond selectively to aversive signals. And despite finding that optogenetic activation of VP glutamate terminals led to monosynaptic EPSCs in a large majority of the VTA neurons assessed, we detected no VTA Fos induction following their in vivo activation. We did however observe Fos induction in the LHb that is consistent with the strong avoidance behavior evoked by activation of VGLUT2+ VP terminals in LHb^40^. Altogether, our data are generally consistent with the notion that VP GABA neurons produce reinforcement, and VP glutamate neurons drive avoidance, by a preferential functional targeting of VTA interneurons, and excitatory projection neurons in LHb. However, additional experiments will be required to ratify and expand on the precise circuit mechanisms, particularly in VTA where GABA and glutamate projection neurons impact reward/avoidance behaviors independent of local effects on dopamine neurons^50,51,55^.

Prior studies showed that VP cells fire between 2 and 20 Hz under basal conditions in behaving rats, with bursts between 10 and 50 Hz in response to reward and reward-predicting cues^2,16,17,19,36,37^. A recent study from Yang and collaborators also reported that VGLUT2^+^ neurons of the basal forebrain were able to fire up to 80 Hz ex vivo^56^. In our study, high-frequency cell type-specific stimulation (40 Hz) of either VP GABA or glutamate neurons evoked the strongest behavioral responses, consistent with earlier studies using nonspecific high-frequency electrical stimulation^9,57^. High-frequency stimulation at the level of projection target recapitulated behaviors when stimulating glutamate terminals in the LHb and VTA, and GABA terminals in the VTA, but not GABA terminals in the LHb. These data are consistent with our anatomical data, and strongly support the hypothesis that VP glutamate inputs to LHb and VP GABA inputs to VTA drive much of the avoidance and appetitive behaviors, respectively. However, caveats apply. For example, we can’t exclude the possibility that optogenetic activation of VP terminals antidromically activates VP neurons that project elsewhere. Though our observation that VP neurons rarely collateralize to both LHb and VTA mitigates this concern. Alternatively, optogenetic stimulation of VP projections to LHb and VTA may also activate nearby or passing fibers, for example, in the mediodorsal thalamus or to the PPTg, respectively. Future experiments using intersectional genetic approaches may provide additional insight.

Previous reports have demonstrated the presence of transcripts encoding VGLUT2 localized to cells within the VP^30^. We used reporter mouse to quantitatively map the proportion of VP glutamate cells relative to GABA or cholinergic cells. We found the glutamate neurons concentrated in the VPvm and in sections rostral to the midline crossing of the anterior commissure. Thus, previous studies employing anatomical approaches to target rostral versus caudal VP may have enriched for or against, respectively, VP glutamate neurons^25,26^. Indeed, the density of glutamate neurons may relate to the ability of caudal but not rostral VP manipulations to trigger place preference, blunt cocaine-induced reinstatement, or modulate “liking” reactions and consummatory behavior^12,27–29^.

Using retrograde tracers and in situ hybridization, a previous study indicated that VP glutamate neurons project to VTA^32^. We used a Cre-dependent strategy to label VP glutamate neurons and map their projections in comparison with VP GABA and cholinergic cell types. Consistent with previous observations, VP cholinergic terminals preferentially target prefrontal cortex and basolateral amygdala^58,59^, showing a projection pattern distinct from VP glutamate and GABA cells. On the other hand, VP glutamate and GABA share qualitatively very similar afferents, targeting a nearly identical set of brain regions, and in line with a recent report characterizing projections from VGLUT2^+^ basal forebrain cells^35^. In addition, we used a unique approach to simultaneously label glutamate and GABA projections in the same VP region of the same animal, allowing us to compare the density of their relative input to key target structures. Consistent with the functional data, we observed that VP glutamate neurons more heavily and extensively innervate LHb, while the GABA neurons more heavily innervate VTA.

Neurochemical phenotyping of VP glutamate neurons revealed that 12.5% co-expressed the calcium-binding protein PV. These observations are in line with a recent report showing that ~60% of Parva^+^ VP cells were *Gad1*^+^, suggesting the potential for Parva^+^ VP cells to release other neurotransmitters^33^. The cholinergic marker ChAT was virtually absent in VP glutamate neurons. However, we did detect a small fraction of VP glutamate cells co-labeled for the GABAergic marker VGAT. These data may explain our finding that a subset of VGLUT2^+^ VP terminals in the LHb, but not the VTA, appeared capable of GABA co-release. Curiously, we failed to detect reciprocal glutamate release in the LHb when targeting ChR2 to VGAT^+^ neurons. Nevertheless, these data are in line with numerous reports demonstrating glutamate/GABA co-release in the LHb^51,60–63^, indicating that the LHb may be specialized to this form of co-transmission.

In this study, we described the location of glutamate cells and their relative abundance throughout the rostro-caudal axis of the VP, with a dense cluster of cells located in the VPvm. We showed that glutamate and GABA cells project to qualitatively similar targets but to different functional effect. Importantly, we revealed that both excitatory and inhibitory VP cells can drive motivated behavior, and with opposite valences. Fine tuning of these inhibitory–excitatory signaling pathways could be critical for normal hedonic and motivational processes. Future investigation should focus on modifications occurring at the level of VP neuron types in the neuropathology associated with drug addiction and neuropsychiatric disorders.

## Methods

### Animals

Homozygous breeders for VGLUT2-IRES-Cre, VGAT-IRES-Cre, and ChAT-IRES-Cre knock-in mice^64^ were obtained from The Jackson Laboratory: *Slc17a6*^*tm2(cre)Lowl*^ (stock no: 016963), *Slc32a1*^*tm2(cre)Lowl*^ (stock no: 016962), and *Chat*^*Cre*^ (stock no: 017259). BAC transgenic VGLUT2-EGFP mice^65^ were obtained from GENESAT through the MMRRC: *Slc17a6*-EGFP (# 011835-UCD). VGLUT2-Cre and VGLUT2-EGFP mice were fully backcrossed to C57Bl/6; VGAT-Cre and ChAT-Cre mice were mixed 129 Sv × C57Bl/6. The *Gad1*-GFP transgenic mouse line was generously provided by Dr. Yanagawa (Japan)^66^. Mice were group housed and maintained on a 12 h light-dark cycle (i.e., light cycle; 7 am–7 pm) with food and water available ad libitum unless noted. Both male and female mice between 6 and 14-weeks old were included and all experiments were conducted during the light phase of the cycle. All protocols were approved by the University of California San Diego Institutional Animal Care and Use Committee.

### Stereotactic surgery

For intracranial injections, mice (>4 weeks) were deeply anesthetized with isoflurane, placed into a stereotaxic apparatus (Kopf), and 150–250 nl of AAV1-EF1α-DIO-ChR2:YFP (4 × 10^12^ genomes/ml, UNC gene therapy center), AAV5-EF1α-DIO-YFP (6.5 × 10^12^ genomes/ml, UNC gene therapy center), AAV1-EF1α-DIO-ChR2:mCherry (2 × 10^12^ genomes/ml, UNC gene therapy center), AAV5-EF1α-DIO-eNpHR3.0:YFP (3 × 10^12^ genomes/ml, UNC gene therapy center), AAVDJ-EF1α-DO-DIO-tdTomato-eGFP (1.7 × 10^12^ genomes/ml, Salk institute virus vector core) or AAVDJ-hsyn-DIO-mRuby2-P2A-Synaptophysin-EGFP (provided by Dr. B.K. Lim^34,67^) was infused unilaterally into the left VP (LM = −1.45, AP = +0.55, DV = −5.35; mm relative to Bregma), or bilaterally when noted, at 100 nl/min (WPI UltraMicroPump) using custom made beveled 30-gauge stainless (Plastics One, VA) injectors (as described in refs. ^51,54^). rAAV2-retro-pSyn-Cre (4.2 × 10^12^ genomes/ml, Janelia Research) and green retrobeads (Lumafluor) were similarly infused unilaterally into the VTA (LM = −0.4, AP = −3.4, DV = −4.4) and red beads (Lumafluor) unilaterally in the LHb (LM = −0.4, AP = −1.7, DV = −2.8). The injection tip was left in place for an additional 8 min, withdrawn ~0.05 mm, left in place an additional 2 min, then slowly retracted. For mice used in behavioral experiments, following viral infusion mice were implanted with an optic fiber constructed from 200-µm core multimode optical fiber (FT200EMT, Thorlabs) inserted into a ceramic ferrule (as described in refs. ^51,67,68^) at one of the following coordinates (in mm relative to Bregma): VP (LM = −1.45, AP = 0.55, DV = −4.5), LHb (LM = −0.7, AP = −1.58, DV = −1.7), or VTA (LM = −0.5, AP = −3.4, DV = −4.0). Fibers were stabilized in place using dental cement (Lang dental) secured by two skull screws (Plastics One). Animals were treated with analgesic Carprofen (Pfizer, 5 mg/kg s.c.) prior to and the day after surgery. Mice were monitored daily and allowed to recover from surgery >3 weeks prior to subsequent behavioral or physiological assays.

### Histology

Mice were deeply anesthetized with a mixture of ketamine (Pfizer, 10 mg/kg i.p.) and xylazine (LLOYD, 2 mg/kg i.p.) and transcardially perfused with 10 ml of phosphate buffered saline (PBS) followed by ~50 ml 4% paraformaldehyde (PFA) at a rate of 5–6 ml/min. Brains were extracted, post-fixed in 4% PFA at 4 °C overnight, and transferred to 30% sucrose in PBS for >48 h at 4 °C. Brains were frozen in isopentane and stored at −80 °C. For virus expression and optic fiber implant site verification, 30-µm coronal cryo-sections were cut using a cryostat (CM3050S, Leica) and collected in PBS containing 0.01% sodium azide. For immunostaining, brain sections were gently rocked 3 × 5 min in PBS, 3 × 5 min in PBS containing 0.2% Triton X-100 (PBS-Tx), and blocked with 4% normal donkey serum (NDS) in PBS-Tx for 1 h at room temperature (RT). Sections were then incubated in one or more primary antibody: rabbit anti-GFP, 1:2000, Invitrogen A11122; chicken anti-GFP, 1:2000, Invitrogen A10262; rabbit anti-TH, 1:2000, Millipore AB152; sheep anti-TH, 1:2000, Pel-Freez P60101-0; rat anti-substance P, 1:400, Millipore MAB356, goat anti-ChAT, 1:400, Millipore AB144P; mouse anti-parvalbumin, 1:2000, Millipore MAB1572; rabbit anti-calbindin, 1:400; Millipore AB1778; mouse anti-calbindin, 1:400, Swant McAB300; rabbit anti-neurotensin, 1:1000, Immunostar 20072; rabbit anti-Fos, 1:1400, Cell Signaling 2250 s, guinea pig anti-NeuN, 1:3000, Millipore ABN90 in block at 4 °C overnight. Sections were rinsed 3 × 10 min with PBS-Tx and incubated in appropriate secondary antibodies (Jackson ImmunoResearch) conjugated to Dylight405, Alexa488, Alexa594 or Alexa647 fluorescent dyes (5 µg/ml) for 2 h at RT. Sections were washed 3 × 10 min with PBS, mounted on slides, and coverslipped with Fluoromount-G mounting medium (Southern Biotech) ± DAPI (Roche, 0.5 µg/ml).

### Fluorescent in situ hybridization

VGLUT2-EGFP mice were killed by cervical dislocation and fresh brains were extracted and frozen in isopentane before storage at −80 °C. Twenty-micrometer coronal cryo-sections were cut using a cryostat (CM3050S, Leica) and placed directly onto superfrost slides (Thermo scientific) before air-tight storage at −80 °C. In situ hybridizations were performed using the RNAscope Multiplex Fluorescent Assay (advanced cell diagnostics). *Slc17a6* (VGLUT2; ref 319171), *GFP* (ref 400281), and *Slc32a1* (VGAT; ref 319191) probes were couples to alexa 488, atto 550, and atto 647, respectively. DAPI was used to label nuclei and identify cells.

### Imaging and cell counting

Cell counts and histochemical characterization were performed on images acquired using a Zeiss AxioObserver Z1 widefield epifluorescence microscope (10 × 0.45 NA, 20 × 0.75 NA, or 63 × 1.4 NA objective) and Zen blue software, or on images acquired with NanoZoomer 2 HT (20 × 0.75 NA objective with ×2 lens converter) plus fluorescence module L10387-03 (Hamamatsu). High-magnification display images were acquired with a Zeiss ApoTome 2.0 for structured illumination. VP boundaries were defined using Substance P staining. Mice were excluded of behavioral studies when spreading was observed in the lateral preoptic area (LPO) [known to project to both LHb and VTA^69,70^], bed nucleus of the stria terminalis (BNST) or NAc or when misplacement of the optic fiber was detected. Cell counting was conducted manually using the NDP viewer for Nanozoomer 2 HT images and/or the cell counter plugin of ImageJ for Zeiss AxioObserver images on one 30-µm section every 150 µm along the rostral caudal extent of the VP from Bregma +1.34 to −0.22 mm. C-fos counting in the VTA and LHb was performed manually by a blinded experimenter on four 30-µm sections per brain, every 150 µm, from Bregma −1.34 to −1.94 mm (LHb) and −3.16 to −3.8 mm (VTA). Counting of *Slc17a6*, *GFP* and *Slc32a1* mRNA positive-cells was done on apotome-illumination ×63 magnification z-stack images sampled in the VP of brain sections from Bregma +0.38 and +0.26 mm. DAPI counterstain was used to define cells. Cells were defined as positive for a certain mRNA presence when presenting at least four clear fluorescent puncta surrounding DAPI staining.

### 2-nosepoke ICSS task

Prior to the first day of testing mice were food-restricted overnight and subsequently provided restricted access for 3 h daily at the end of each session to facilitate behavioral responding. At the beginning of the session, the ferrule was connected to a 50-µm optical patch cable through an optical commutator (Doric Lenses, Canada) and mice were placed in operant chambers (Med associates) controlled by MedPC IV software. The start of the 60-min session (or 30-min session when noted) was signaled by a brief tone (2 kHz, 0.5 s) and illumination of overhead house light and LED cue lights over the nosepoke holes. The chamber contained two photobeam-equipped nosepoke holes which were each baited at the start of each session with a sucrose pellet (Bio-Serv, F0071). Beam-breaks on the active nosepoke led to: a 0.5 s tone, the LED cue lights over the nosepokes turned off for 2 s, and the activation of a TTL-controlled DPSS laser (473 nm, Shanghai or OEM laser) set to deliver 10 mW (80 × mW/mm^2^ at 200 µm fiber tip) pulses at 40 Hz (1 s) with a 10-ms pulse width controlled by Master-8 (A.M.P.I.) or Arduino stimulus generators. Nosepokes that occurred during the 2 s light off were recorded but without effect. Inactive nosepokes led to identical tone and cue light effects but did not trigger the laser. Laser power was measured using a digital power meter (Thorlabs PM100D/S121C). Mice were tested over 3 days. Active and inactive nosepokes were switched on a fourth testing day to assess for potential side bias.

### Fos measurement

Fos expression was measured following two different protocols. Mice were placed in operant chambers for a 30-min ICSS protocol as previously described or a passive stimulation protocol in which nosepokes did not trigger any stimulation, but where animals received 1-s stim every 5 s for a total of 360 stim in 30 min. This pattern of stimulation was selected to compare the average number of stim mice would work for in 30 min. Mice were then anesthetized, transcardially perfused with PFA 4% and brain extracted 90 min after beginning of the experiment.

### 5-choice nosepoke ICSS task

Mice were food restricted and tethered to the patch cable as described for the ICSS task. Identical chambers, lasers, and conditions were used but a five-nosepoke bar (Med Associates) was inserted in place of the two nosepoke holes. Each of five-nosepoke holes led to a 1-s stimulation, but each were assigned a variable frequency (0, 5, 10, 20, and 40 Hz) or pulse duration (2, 5, 10, 15, and 20 ms). All other parameters were as described in the ICSS procedure.

### Real-time place preference/avoidance

On a baseline (pre-test) day, mice were placed on the border between two adjoining (20 × 20 cm) homogenous gray compartments and the amount of time spent in each compartment was recorded using video tracking software (Anymaze). Most mice displayed no preference, but those with >75% side preference on pre-test were excluded from further study. On the subsequent day, one side was designated active and entry to the active side triggered photostimulation (40 Hz, 10 ms pulse width, 10 mW), using the lasers as described above but controlled by an ANY-maze interface (Stoelting). For inhibition of VP cell bodies, mice were tethered to a green DPSS laser (532 nm, Shangai laser) and received 10 mW continuous stimulation when located in the active side of the apparatus. Sessions lasted for 20 min and the amount of time spent in each compartment, distance traveled, speed, and number of crossings were recorded. For Halo inhibition, a 7-day protocol was used consisting of a pre-test day, 3 days of test (side A), and 3 days of “switch” (side B). The data presented on Fig. 5c, f are averages of the third day of test (side A) and third day of switch (side B) phases.

### Conditioned place-preference

On pre-test day mice were placed on the border between two adjoining white (20 × 20 cm) and black (20 × 20 cm) compartments with different floorings (large vs. small mesh), different odors beneath the floorings (cloves vs. ginger), and allowed free access for 20 min. Pre-test was followed by a 3 day-conditioning phase, with two sessions per day, during which animals were confined in one or the other compartment for 20 min. One compartment was assigned as the stimulation-associated side and the other as the non-stimulation-associated side to equilibrate the average time spent in each compartment in all groups during pre-test. Mice were tethered as described above during conditioning sessions. During the conditioning sessions, mice received 40 Hz, 10-ms pulse width, 10-mw stimulation throughout the 20-min session. Stimulation and non-stimulation sessions were randomized between morning (AM) and afternoon (PM), with at least 6 h separation. Post-test was performed on day 5 identical to pre-test. Time in each compartment was measured as previously and time spent in the stimulation-associated compartment was compared between pre-test and post-test.

### Electrophysiological recordings

Adult mice (6–12 weeks) were deeply anesthetized with pentobarbital (200 mg/kg i.p.; Virbac) and perfused intracardially with 10 ml ice-cold sucrose-artificial cerebrospinal fluid (ACSF) containing (in mM): 75 sucrose; 87 NaCl, 2.5 KCl, 7 MgCl_2_, 0.5 CaCl_2_, 1.25 NaH_2_PO_4_, 25 NaHCO_3_ and continuously bubbled with carbogen (95% O_2_–5% CO_2_). Brains were extracted and 200-μm coronal slices were cut in sucrose-ACSF using a Leica Vibratome (vt1200). Slices were transferred to a perfusion chamber containing ACSF at 31 °C (in mM): 126 NaCl, 2.5 KCl, 1.2 MgCl_2_, 2.4 CaCl_2_, 1.4 NaH_2_PO_4_, 25 NaHCO_3_, 11 glucose, continuously bubbled in carbogen. After >60 min recovery, slices were transferred to a recording chamber continuously perfused with ACSF (1–3 ml/min). Patch pipettes (3.5–6.5 MΩ) were pulled from borosilicate glass (King Precision Glass) and filled with internal recording solution containing (in mM): 120 CsCH_3_SO_3_, 20 HEPES, 0.4 EGTA, 2.8 NaCl, 5 TEA, 2.5 Mg-ATP, 0.25 Na-GTP, at pH 7.25 and 285 ±5 mOsm. For current-clamp recordings of ChR2 spike fidelity and eNpHR3.0 inhibition experiments, potassium-based recording solution was used (in mM): 123 CH_3_KO_3_S, 10 HEPES, 0.2 EGTA, 8 NaCl, 2.5 Mg-ATP, 0.25 Na-GTP, at pH 7.25 and 280 ± 5 mOsm.

mCherry-labeled VP neurons and terminals were visualized by epifluorescence and visually guided patch recordings were made using infrared-differential interference contrast (IR-DIC) illumination (Axiocam MRm, Examiner.A1, Zeiss). ChR2 was activated by flashing blue light (473 nm; 5-ms pulse width to trigger post-synaptic currents in whole cell, applied at 40 Hz pulse train for 5 s in cell-attached recordings) through the light path of the microscope using an ultrahigh-powered light-emitting diode (LED460) (Prizmatix) under computer control. Excitatory/inhibitory post-synaptic currents (E/IPSCs) were recorded in whole-cell voltage clamp. Action potentials were recorded in whole-cell current clamp (*I* = 0) or cell-attached modes (Multiclamp 700B amplifier, Axon Instruments), filtered at 2 KHz, digitized at 10 KHz (Axon Digidata 1550, Axon Instruments), and collected on-line using pClamp 10 software (molecular device). Series resistance and capacitance were electronically compensated prior to recordings. Liquid-junction potential, which estimated to be 12 mV, was not corrected. Series resistance and/or leak current were monitored and cells that showed >25% change during recordings were defined as unstable and discarded. Neurons were held in voltage-clamp at −65 mV to record AMPAR EPSCs and at 0 mV to record GABA_A_R IPSCs in whole-cell configuration. For whole-cell voltage-clamp recordings, single-pulse (5 ms) photostimuli were applied every 45 s and 10 photo-evoked currents were averaged per neuron per condition. For cell-attached studies on firing rate, photostimuli trains were delivered every 60 s and three responses averaged per neuron where action potential frequency was averaged over the 5 s before, during, and after the 5-s stimulation train. For spike fidelity study, action potentials were counted for every single pulse over the 1 or 5 s of stimulation. eNpHR3.0 was activated by flashing green light (532 nm): 5-ms pulse width to record single-inhibitory currents, and 1-s pulse width during a 3-s positive current injection step to elicit neuronal firing blockade. DMSO stock solutions of drugs were diluted 1000-fold in ACSF and bath applied at the following concentrations: DNQX (10 μM, Sigma), Picrotoxin (10 μM, Sigma). Current sizes were calculated by using peak amplitude from baseline.

### Statistics

To evaluate statistical significance, data were subjected to two tailed Student’s *t*-tests, RM one or two-way ANOVAs followed by Sidak post hoc analysis, or RM three-way ANOVAs followed by Tukey–Kramer post hoc analysis when appropriate (GraphPad prism v6 or Statistica v.6.1). Non-parametric Wilcoxon matched-pairs signed-rank tests were preferred to compare AP frequencies in Figs. 6 and 7. Statistical significance was set at *p* < 0.05. All data are presented as means ± SEM unless noted. See Table S2 for detailed statistical information.

### Data availability

The authors declare that all data supporting the findings of this study are available within the paper and its supplementary information files.

## Electronic supplementary material

Supplementary Information
