# Supplementary material for: Opponent control of behavioral reinforcement by inhibitory and excitatory projections from the ventral pallidum
Source: Nat Commun. 2018 Feb 27;9:849. doi: 10.1038/s41467-018-03125-y (PMC5829073; doi:10.1038/s41467-018-03125-y)
Supplement: Supplementary file 1 — Supplementary Information [file 41467_2018_3125_MOESM1_ESM.pdf]

Opponent control of behavioral reinforcement by inhibitory and excitatory projections from the ventral pallidum

Faget et al.

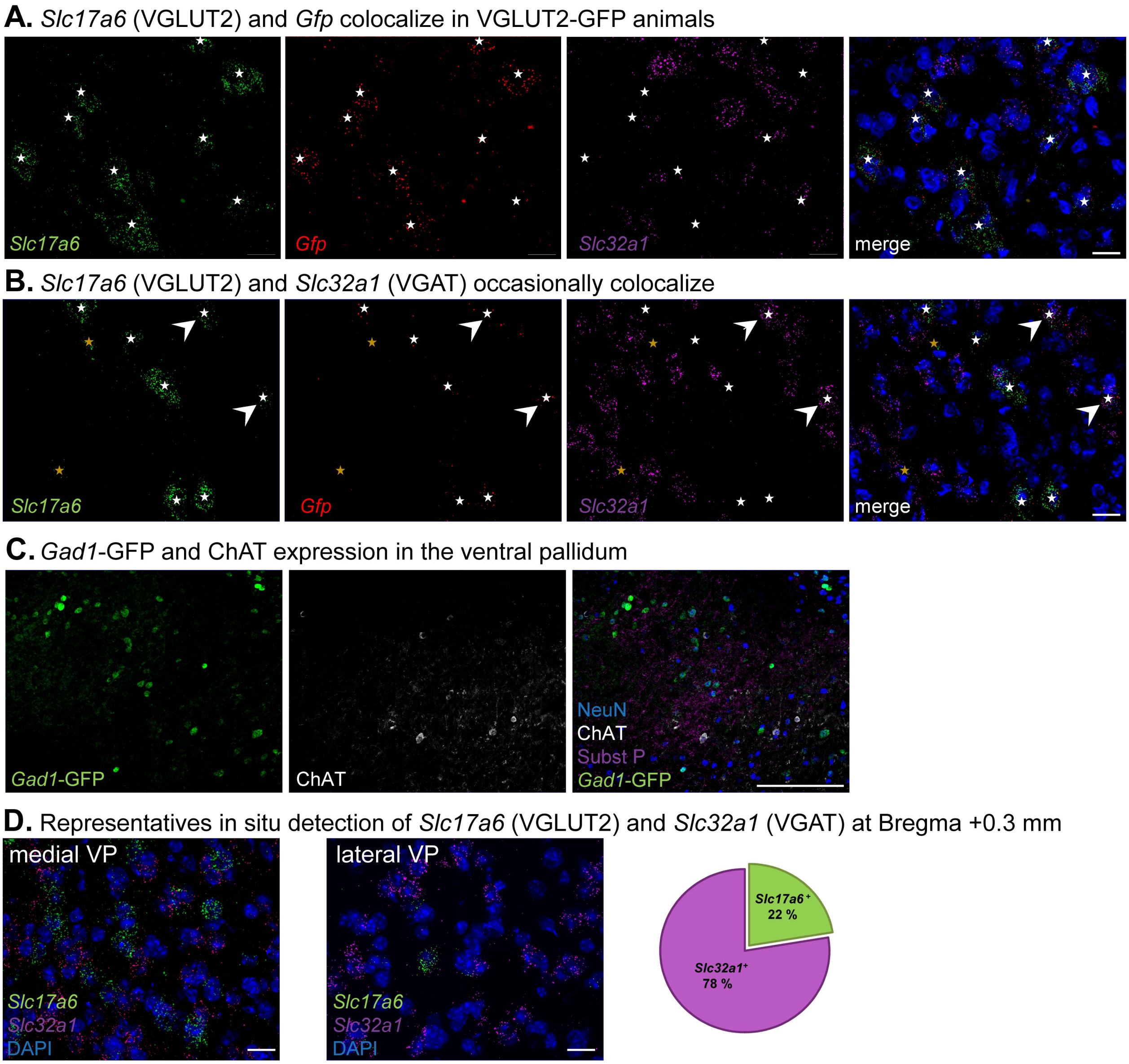

Supplementary Figure 1. Related to Figure 1.
In situ detection of *Slc17a6* (VGLUT2), *Slc32a1* (VGAT) and *Gfp* mRNA in the VP of VGLUT2-GFP mice. A. Representative image of labeled VP sections showing colocalization (white stars) of *Slc17a6* (VGLUT2, green) with *Gfp* (red). *Slc32a1* (VGAT, purple) generally does not colocalize. **B.** Occasionally we identified putative *Slc17a6* labeled cells that did not clearly label with *Gfp* probes, though the signal was generally weaker (yellow stars). Occasional colocalization between *Slc17a6* and *Slc32a1* was also observed (white arrowheads), consistent with Fig. 1D. DAPI (blue) **C.** Representative image of GFP expression and ChAT immunostaining in the VP of *Gad1*-GFP animals. Substance P (purple) and NeuN (blue). **D.** Representative in situ detection of *Slc17a6* (VGLUT2, green) with *Slc32a1* (VGAT, purple) in the medial and lateral VP at Bregma +0.38 mm where *Slc17a6* represent 22 % and *Slc32a1* 78 % of *Slc17a6* and *Slc32a1* total cell counts. Scales= 20 µm (A, B and D) and 200 µm (C).

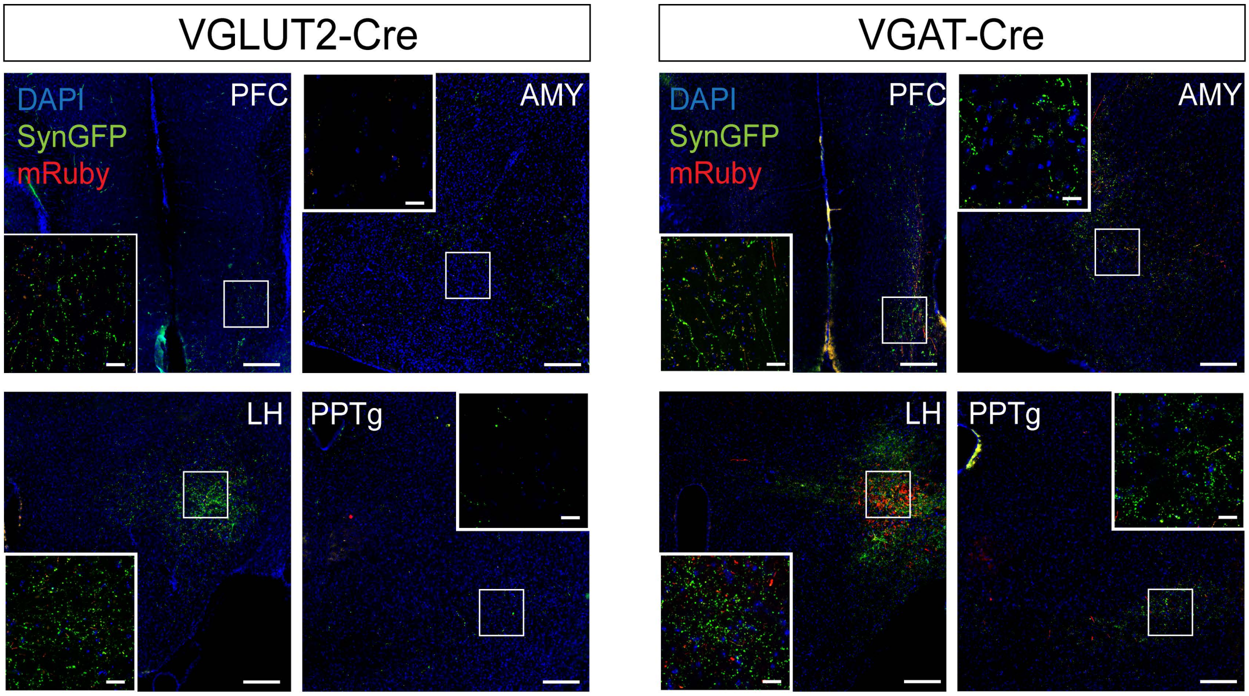

Supplementary Figure 2. Related to Figure 2.
VP glutamate and GABA neuron projections to other brain regions. Expression of mRuby (red) in processes and Synaptophysin:GFP (SynGFP, green) in synaptic terminals of VP glutamate and GABA cells using VGLUT2-Cre and VGAT-Cre mouse lines. $VGLUT2^{+}$ and $VGAT^{+}$ VP terminals were detected in the prefrontal cortex (PFC), medial amygdala (AMY), lateral hypothalamus (LH) and pedunculopontine nucleus (PPTg). Scales= 200 μm and 20 μm (insets).

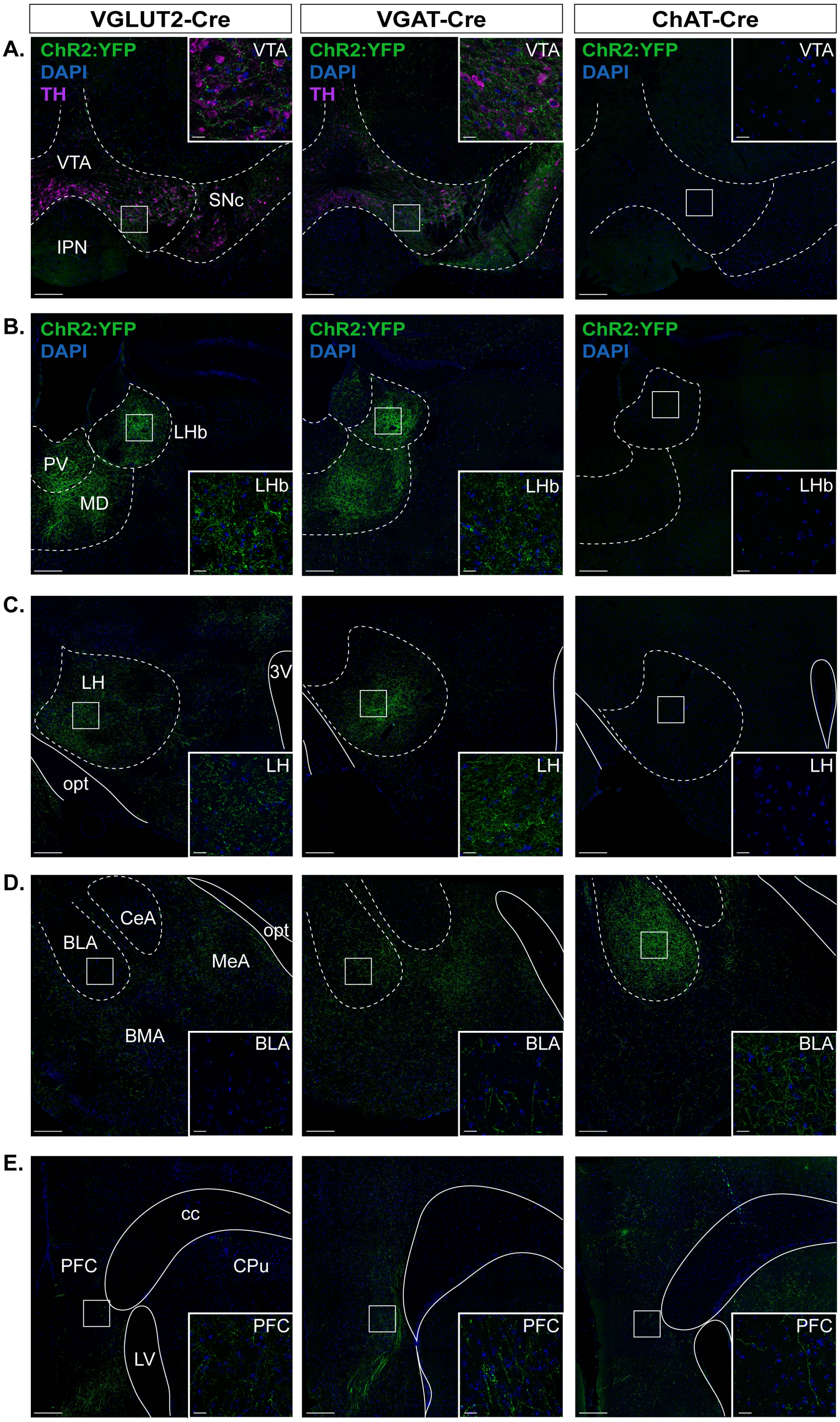

Supplementary Figure 3. Related to Figure 2.

Projections of VP neurons by neurotransmitter-defined cell type. Expression of ChR2:YFP (green) in putative terminals of VP glutamate, GABA, and acetylcholine neurons using VGLUT2-Cre, VGAT-Cre, or ChAT-Cre mouse lines. VP glutamate and GABA neurons projected to similar targets in the **A.** ventral tegmental area (VTA) and substantia nigra pars compacta (SNc), **B.** lateral habenula (LHb) and medio-dorsal thalamus (MD), **C.** lateral hypothalamus (LH), D. medial amygdala (MeA) and E. prefrontal cortex (PFC). Cholinergic VP neurons projected more narrowly targeting the **D.** basolateral amygdala (BLA) **E.** and PFC. IPN: interpeduncular nucleus, PV: paraventricular nucleus of the thalamus, opt: optic tract, 3V: third ventricle, CeA: central amygdala, BMA: baso-medial amygdala, cc: corpus callosum, LV: lateral ventricle, CPu: Caudate Putamen. Scale= 200 μm (widefield), and 20 μm (insets).

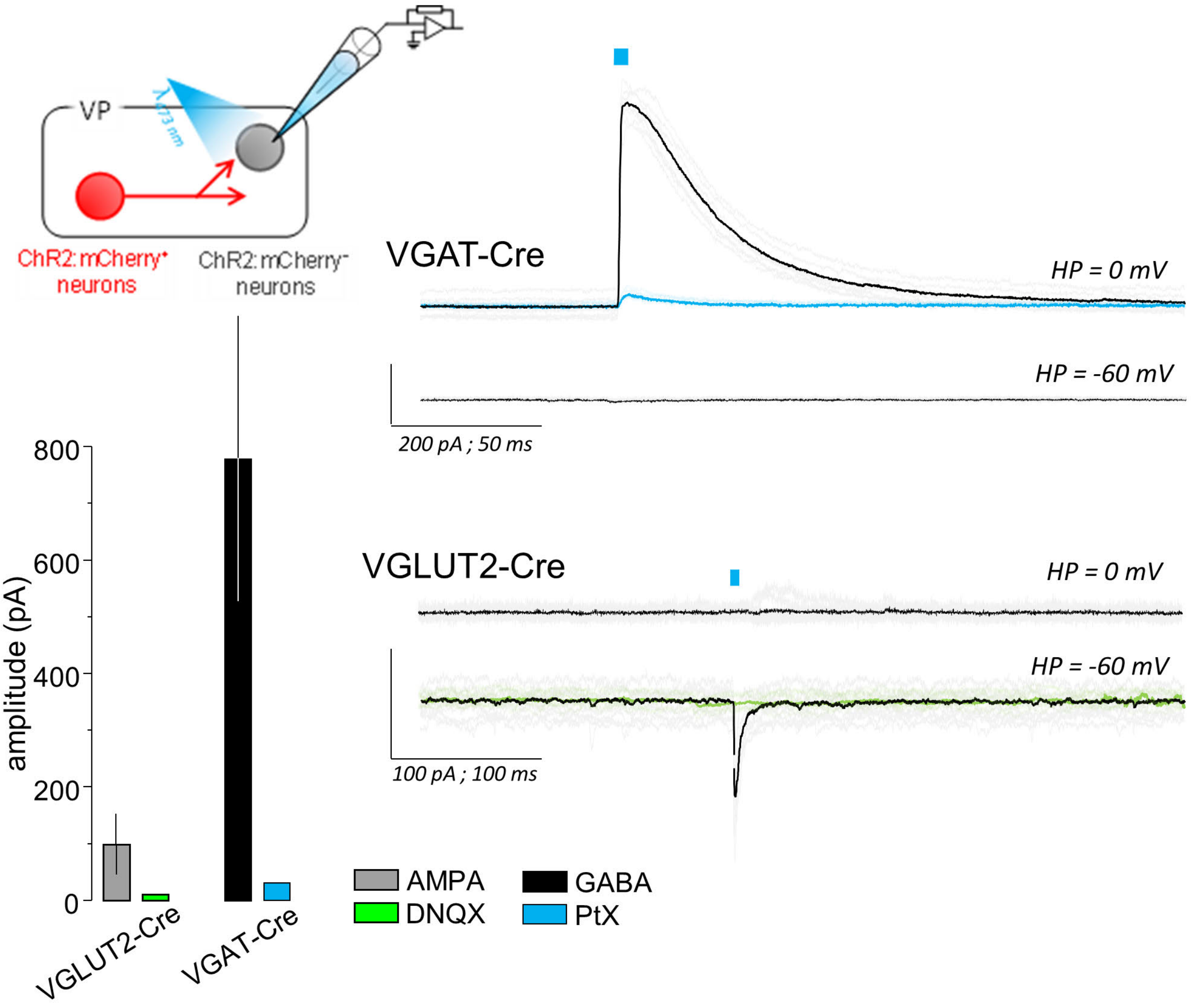

Supplementary Figure 4. Related to Figure 2.

VGAT+ and VGLUT2+ VP neuron stimulations show local GABA and glutamate connectivity.
ChR2:mcherry was expressed in VGLUT2$^+$ or VGAT$^+$ VP neurons and recordings were made from mCherry negative VP neighbors. Single-pulse (5-ms, blue dashes) photostimulation of VGLUT2$^+$ VP fibers triggered DNQX-sensitive EPSCs recorded at -60 mV. While stimulation of VGAT$^+$ VP fibers triggered PTx-sensitive IPSCs recorded at 0 mV. Bar graphs show peak amplitude of recorded EPSCs (n=6; DNQX n=2) and IPSCs (n = 6; PtX n = 1). Inset shows representative glutamate and GABA postsynaptic currents. Scale IPSC = 200 pA; 50 ms; Scale EPSC = 100 pA; 100 ms.

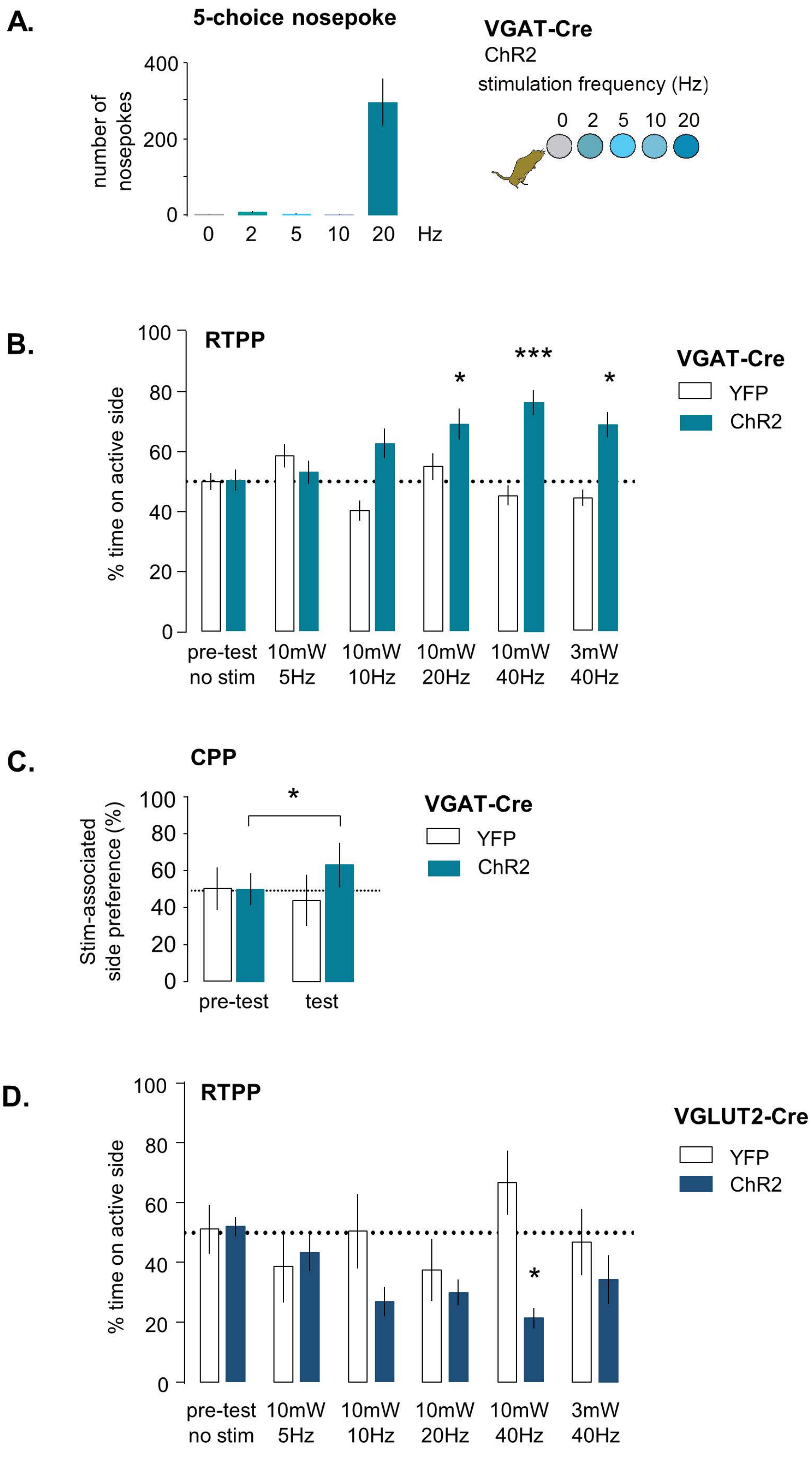

Supplementary Figure 5. Related to Figures 3 and 4.
Comparison across light frequencies and intensities in the RTPP assay. A. In a 5-choice nosepoke ICSS task for VP GABA neuron stimulation mice preferred 20-Hz stimulation when this was the highest frequency presented. **B.** Percent of time spent in the stimulation-paired compartment during RTPP in response to stimulation of VGAT^{+} cells in the VP (n= 8 VGAT control, n= 10 VGAT ChR2, RM 2-way ANOVA, viral treatment x stim parameters, $F_{(5,80)}$ = 6.3, p < 0.0001). The same animals were tested daily across the conditions noted. **C.** Percent of time spent in the stimulation-associated compartment during the pre- and post-tests of a conditioned place preference (CPP) assay; (n= 6 VGAT control, n= 9 VGAT ChR2, RM 2-way ANOVA, viral treatment x day, $F_{(1,13)}$ = 6.2, p = 0.03). **D.** Percent of time spent in the stimulation-paired compartment during the RTPP in response to stimulation of VGLUT2^{+} cells in the VP (n= 8 VGLUT2 control, n= 12 VGLUT2 ChR2, RM 2-way ANOVA, viral treatment x stim parameters, $F_{(5,90)}$ = 2.7, p = 0.03). *p< 0.05, ***p< 0.001.

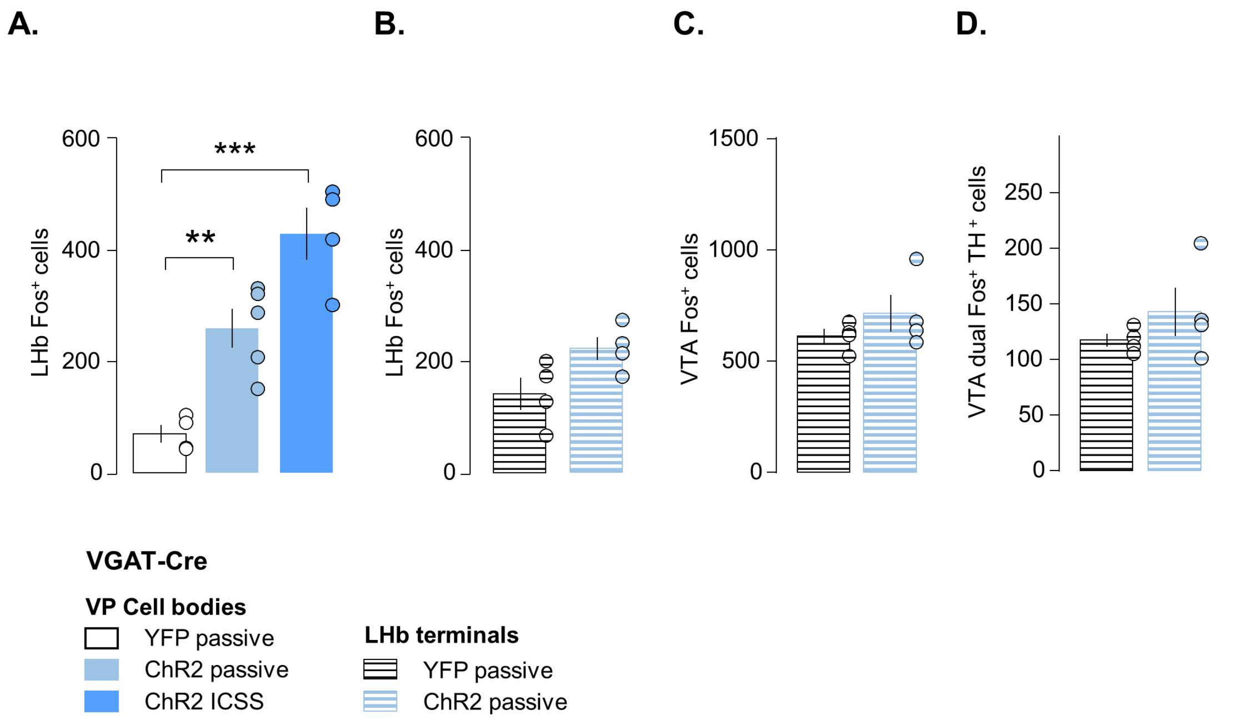

Supplementary Figure 6. Related to Figure 3.
Fos expression in the LHb after VP GABA cell bodies or LHb-projecting fibers stimulation. A. Fos^+ cells in the LHb increased following VP GABA neurons activation (n = 4 VGAT YFP and ChR2 ICSS, n = 5 VGAT ChR2 passive; unpaired *t*-test; VGAT YFP vs. ChR2 passive; $t_{(7)} = 5.2$, p = 0.001; VGAT YFP vs. ChR2 ICSS, $t_{(6)} = 7.9$, p = 0.0002). However, stimulation of VP GABA terminals in the LHb did not induce an increase in Fos^+ positive cell numbers in **B.** the LHb (n = 4, unpaired *t*-test; YFP passive vs. ChR2 passive; $t_{(6)} = 2.3$, ns) or **C-D.** the VTA (n = 4, unpaired *t*-test; YFP passive vs. ChR2 passive; Fos^+ cells, $t_{(6)} = 1.157$, ns; Fos^+ TH^+ cells, $t_{(6)} = 1.145$, ns) . **p<0.01, ***p<0.001.

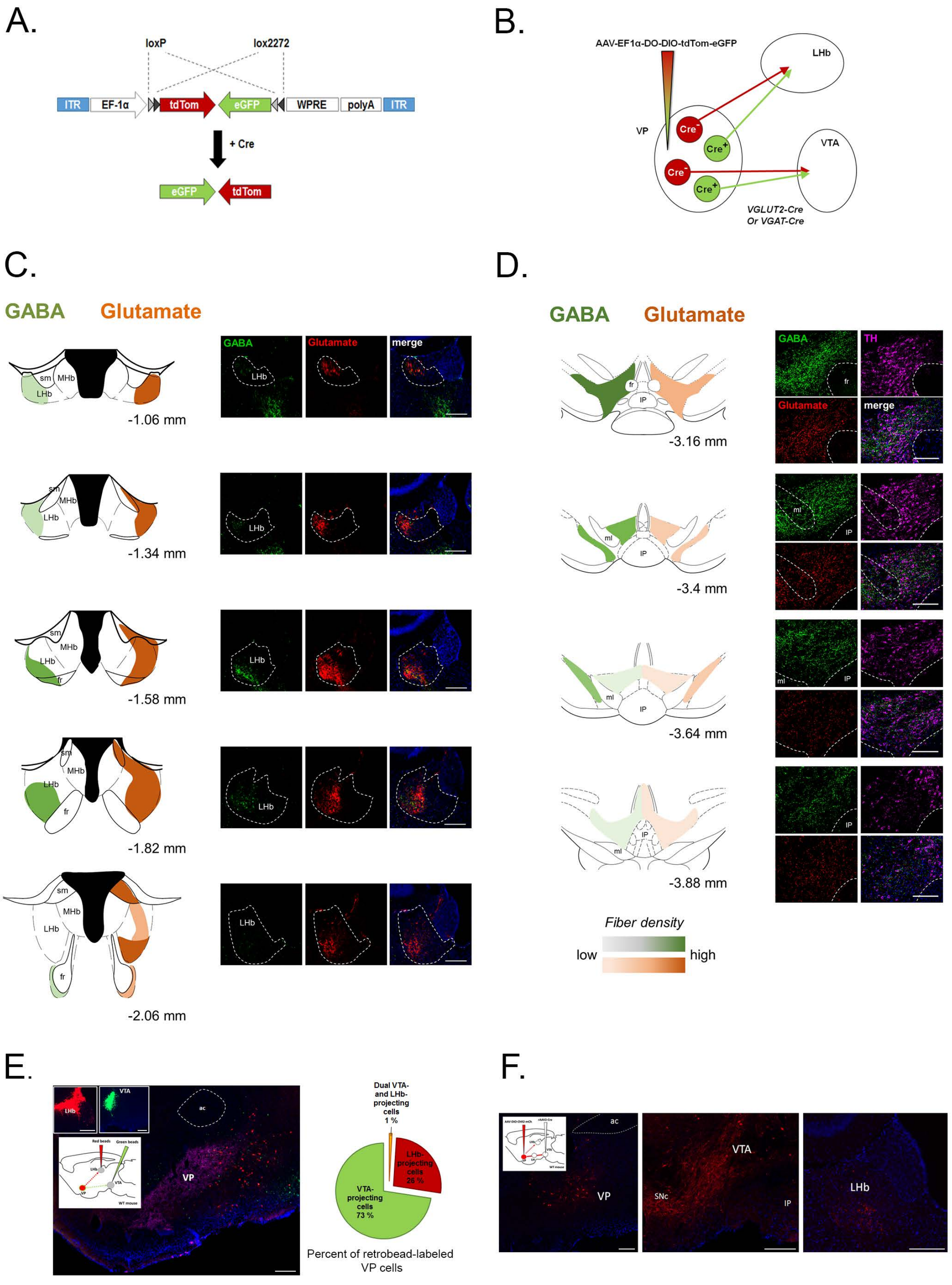

Supplementary Figure 7. Related to Figures 6 and 7.
Simultaneous sub-structural mapping of VP glutamate and GABA terminals in the LHb and VTA and rare collateralization of VP neurons to both VTA and LHb. A. AAV-DO-DIO cassette to **B.** simultaneously label Cre$^+$ and Cre$^-$ neurons with different fluorophores in the same animals. Localization and density of fibers expressing GFP (VGAT$^+$ projections) and tdTomato (VGLUT2$^+$ projections) in **C.** LHb and **D.** VTA. Representative images are from a VGAT-Cre animal. Intensity of colors (green or orange) is proportional to the density of fibers. **E.** Different colored retrobeads injected into the LHb (red) and VTA (green) led to only very low levels of colocalization in VP, 1 % (n = 2 mice). **F.** RetroAAV-Cre was injected into VTA, and AAV-DIO-ChR2:mCherry into VP; mCherry signal is abundant in VP and VTA but sparse in LHb. Scale= 200 µm.

Supplementary Table 1. Related to Figure 2. Intensity of VP afferents by cell type and projection target.

		VP projection neurons		
**Abbreviation**	**structure name**	**Glutamate**	**GABA**	**Acetylcholine**
AcbC	accumbens nucleus, core	0	+	+
AcbSh	accumbens nucleus, shell	+/-	+/-	+
AO	Anterior Olfactory Cortex	+	+/-	0
BLA	baso-lateral amygdaloid nucleus	+/-	+/-	+++
BMA	baso-medial amygdaloid nucleus	0	+/-	0
CeA	central amygdaloid nucleus	0	0	0
Den/Ven	dorsal/ventral endopiriform cortex	++	+/-	0
DpMe	Deep Mesencephalic nucleus	0	+	0
DR	dorsal raphe nucleus	+/-	+	0
LDtg	laterodorsal tegmental nucleus	+/-	+	0
LH	Lateral Hypothalamus	++	+++	0
LHb	Lateral Habenula	+++	++	0
MD / PV	Mediodorsal / Paraventricular nuclei of the Thalamus	+++	++	0
MEA	medial amygdaloid nucleus	+	++	+
MHb	Medial Habenula	+/-	+/-	0
MnR	median raphe nucleus	+/-	+	0
mPFC	medial prefrontal cortex (PrL, IL, DP, DTT)	+/-	+	++
PAG	periaqueductal gray	+/-	+/-	0
PF	Parafasicular Thalamic Nucleus	+	+	0
PPtg	pedunculopontine tegmental nucleus	+/-	+	0
SNc	Substantia Nigra Compacta	+	++	0
STh	Sub-Thalamic Nucleus	0	+/-	0
SuM	supramammillary nucleus	+/-	+	0
VTA	Ventral Tegmental Area	++	+++	0

0 indicates no signal detected
+/- indicates scattered terminals were labeled
+ symbols are proportional to relative density of signal within cell type

Supplementary Table 2. Related to all Figures. Statistical procedures and outcomes.

Figure		Description				N-number	Statistic	P value		Post-hoc	Post-hoc P value	
		brain region	genotype	measurement	factors							
1	A	VP	VGLUT2-EGFP	Representative images								
	B		VGLUT2-EGFP	VGLUT2+, GAD67+ and ChAT+ cell counts		9888 cells, 1791 VGLUT2+ cells, 6196 GAD67+ cells, 1901 ChAT+ cells 166 sections, 9 mice						
	C		VGLUT2-EGFP	Representative images								
	D		VGLUT2-EGFP	Representative images								
			VGLUT2-EGFP x VGAT-Cre	Representative image								
			VGLUT2-EGFP - ChAT staining	Percent of VGLUT2+ cells		1078 VGLUT2+ cells, 8 VGLUT2+ ChAT+ cells, 36 sections, 3 mice						
			VGLUT2-EGFP - PV staining	Percent of VGLUT2+ cells		1692 VGLUT2+ cells, 200 VGLUT2+ PV+ cells, 44 sections, 4 mice						
			VGLUT2-EGFP x VGAT-Cre - AAV-DIO-mCherry expression	Percent of VGLUT2+ cells		820 VGLUT2+ cells, 19 VGLUT2+ VGAT mCh+ cells, 16 sections, 3 mice						
2	A	VP coronal section	VGLUT2-Cre VGAT-Cre	Representative images								
	B	sagittal section		Representative images								
	C	sagittal section		Representative images								
	D	VTA coronal section		Representative images								
	E	LHb coronal section		Representative images								
3	A	VP	VGAT-Cre	Representative images & schematic								
	B			AP / stim & example trace		9 cells						
	C			5 choice ICSS	Stimulation frequency	7 ChR2 mice	**Repeated measure (RM) One-way ANOVA**<br>F (4, 24) = 33.16 ; frequency	p < 0.0001	***	**Sidak:** 0 vs. 40Hz<br>**Sidak:** 5 vs. 40Hz<br>**Sidak:** 10 vs. 40Hz<br>**Sidak:** 20 vs. 40Hz	p < 0.0001<br>p < 0.0001<br>p < 0.0001<br>p < 0.0001	***<br>***<br>***<br>***
	D			5 choice ICSS	Stimulation pulse width	7 ChR2 mice	**RM One-way ANOVA**<br>F (4, 24) = 5.617 ; pulse width	p = 0.0025	**	**Sidak:** 2 vs. 10ms<br>**Sidak:** 5 vs. 10ms<br>**Sidak:** 15 vs. 10ms<br>**Sidak:** 20 vs. 10ms	p = 0.006<br>p = 0.003<br>ns<br>ns	**<br>**
	E			2 nspk ICSS	Viral treatment<br>nspk type<br>day	6 YFP mice<br>8 ChR2 mice	**RM 3-way ANOVA**<br>F (1, 24) = 1592 ; viral treatment X nspk type<br>F (2, 48) = 3.471 ; day X viral treatment	p < 0.0001<br>p = 0.04	***<br>*	**Tukey:** day 1 ChR2 active vs. ChR2 inactive<br>**Tukey:** day 2 ChR2 active vs. ChR2 inactive<br>**Tukey:** day 3 ChR2 active vs. ChR2 inactive	p = 0.0001<br>p = 0.0001<br>p = 0.0001	***<br>***<br>***
	F			RTPP	Viral treatment<br>day	9 YFP mice<br>10 ChR2 mice	**RM 2-way ANOVA**<br>F (1, 17) = 7.153 ; viral treatment<br>F (1, 17) = 22.18 ; day<br>F (1, 17) = 14.17 ; day X viral treatment	p = 0.02<br>p = 0.0002<br>p = 0.0015	*<br>***<br>**	**Sidak**: VGAT ChR2 pre-test vs. test	p < 0.0001	***
				RTPP example heat map								
	G	VTA		Fos+ cells	Viral treatment / protocol	4 YFP passive mice<br>5 ChR2 passive mice<br>5 ChR2 ICSS mice	**Unpaired *t* test**<br>t (7)=2.948 ; YFP passive vs. ChR2 passive<br>t (7)=4.437 ; YFP passive vs. ChR2 ICSS	p = 0.02<br>p = 0.003	*<br>**			
				Fos+ TH+ cells	Viral treatment / protocol	4 YFP passive mice<br>5 ChR2 passive mice<br>5 ChR2 ICSS mice	**Unpaired *t* test**<br>t (7)=1.757 ; YFP passive vs. ChR2 passive<br>t (7)=2.601 ; YFP passive vs. ChR2 ICSS	ns<br>p = 0.035	*			
4	A	VP	VGLUT2-Cre	Representative images								
	B			AP / stim & example trace		7 cells						
	C			2 nspk ICSS	Viral treatment<br>nspk type<br>day	5 YFP mice<br>6 ChR2 mice	ns					
	D			RTPP	Viral treatment<br>day	5 YFP mice<br>5 ChR2 mice	**RM 2-way ANOVA**<br>F (1, 8) = 13.84 ; day X viral treatment	p = 0.006	**	**Sidak**: VGLUT2 ChR2 pre-test vs. test	p = 0.017	*
				RTPP example heat map								
	E	VTA		Fos+ cells	Viral treatment	5 YFP passive mice<br>5 ChR2 passive mice	**Unpaired *t* test**<br>t (8)=0.9983 ; YFP passive vs. ChR2 passive	ns				
				Fos+ TH+ cells	Viral treatment	5 YFP passive mice<br>5 ChR2 passive mice	**Unpaired *t* test**<br>t (8)=1.809 ; YFP passive vs. ChR2 passive	ns				
	F	LHb		Fos+ cells	Viral treatment	5 YFP passive mice<br>5 ChR2 passive mice	**Unpaired *t* test**<br>t (8)=5.229 ; YFP passive vs. ChR2 passive	p = 0.0008	***			
5	A	VP	VGAT-Cre	Representative image								
	B			Example trace								
	C			RTPP	Viral treatment<br>day	5 YFP mice<br>6 Halo mice	**RM 2-way ANOVA**<br>F (1, 9) = 9.412 ; day X viral treatment	p = 0.013	*	**Sidak**: VGAT Halo pre-test vs. test	p = 0.014	*
				RTPP example heat map								
	D		VGLUT2-Cre	Representative image								
	E			Example trace								
	F			RTPP	Viral treatment<br>day	5 YFP mice<br>8 Halo mice	**RM 2-way ANOVA**<br>F (1, 11) = 0.8605 ; day X viral treatment	ns				
				RTPP example heat map								
6	A	VTA	VGAT-Cre	IPSCs amplitude	pharmacological treatment	GABA : 19 cells ; 10 mice<br>PTx : 7 cells ; 5 mice	**Paired *t* test**<br>t(6) = 3.18 ; without vs. with PTx	p = 0.019	*			
				Example trace								
	B			AP frequency	stimulation protocol	14 cells ; 8 mice	**Wilcoxon matched-pairs signed rank test**<br>pre-stim vs. during stim	p = 0.0001	***			
				Example trace								
	C			RTPP	Viral treatment	11 ChR2 mice	**Paired *t* test**<br>t (10)=3.086 ; ChR2 pre-test vs. test	p = 0.0115	*			
				Representative image								
	D			2 nspk ICSS	Viral treatment<br>nspk type<br>day	4 YFP mice<br>8 ChR2 mice	**RM 3-way ANOVA**<br>F (1, 20) = 11.84 ; viral treatment X nspk type	p = 0.0026	**	**Tukey:** day 1 ChR2 active vs. ChR2 inactive<br>**Tukey:** day 2 ChR2 active vs. ChR2 inactive<br>**Tukey:** day 3 ChR2 active vs. ChR2 inactive	p = 0.049<br>p = 0.041<br>ns	*<br>*
	E		VGLUT2-Cre	EPSCs amplitude	pharmacological treatment	AMPA : 19 cells ; 7 mice<br>DNQX : 5 cells ; 4 mice	**Paired *t* test**<br>t (4)= 3.75 ; without vs. with DNQX	p = 0.019	*			
				Example trace								
	F			AP frequency	stimulation protocol	14 cells ; 6 mice	**Wilcoxon matched-pairs signed rank test**<br>pre-stim vs. during stim	p = 0.0001	***			
				Example trace								
	G			RTPP	Viral treatment	6 ChR2 mice	**Paired *t* test**<br>t (5)=4.122 ; ChR2 pre-test vs. test	p = 0.0092	**			
				Representative image								

Figure		Description				N-number	Statistic	P value		Post-hoc	Post-hoc P value	
		brain region	genotype	measurement	factors							
7	A	LHb	VGAT-Cre	IPSCs amplitude	pharmacological treatment	GABA : 13 cells ; 8 mice<br>PTx : 7 cells ; 6 mice	**Paired *t* test**<br>t (6)= 2.19 ; without vs. with PTx	p = 0.07	ns			
				Example trace								
	B			AP frequency	stimulation protocol	12 cells ; 7 mice	**Wilcoxon matched-pairs signed rank test**<br>pre-stim vs. during stim	p = 0.0005	***			
				Example trace								
	C			RTPP	Viral treatment	12 ChR2 mice	**Paired *t* test**<br>t (11)=1.862 ; ChR2 pre-test vs. test	p = 0.0896	ns			
				Representative images								
				2 nspk ICSS	nspk type<br>day	8 ChR2 mice	ns					
	D		VGLUT2-Cre	EPSCs amplitude	stimulation protocol	AMPA : 14 cells ; 10 mice<br>DNQX : 6 cells ; 6 mice	**Paired *t* test**<br>t (5)= 4.73 ; without vs. with DNQX	p = 0.005	**			
				Example trace								
				IPSCs amplitude	stimulation protocol	GABA : 5 cells ; 5 mice<br>PTx : 4 cells ; 5 mice	**Paired *t* test**<br>t (3)= 3.77 ; without vs. with PTx	p = 0.033	*			
				Example trace								
	E			AP frequency #1	pharmacological treatment	7 cells ; 5 mice						
				Example trace								
				AP frequency #2	pharmacological treatment	6 cells ; 5 mice						
				Example trace								
	F			RTPP	Viral treatment	6 ChR2 mice	**Paired *t* test**<br>t (5)=3.131 ; ChR2 pre-test vs. test	p = 0.026	*			
				Representative images								

Figure		Description				N-number	Statistic	P value		Post-hoc	Post-hoc P value	
		brain region	genotype	measurement	factors							
S1	A-B & D	VP	VGLUT2-EGFP	Representative images								
	C		GAD67-GFP	Representative images								
S2		PFC, AMY, LH, PPTg	VGLUT2-Cre<br>VGAT-Cre	Representative images								
S3	A-E	VTA, LHb, LH, BLA, PFC	VGLUT2-Cre<br>VGAT-Cre<br>ChAT-Cre	Representative images								
S4		VP	VGLUT2-Cre	EPSCs amplitude	pharmacological treatment	AMPA : 6 cells<br>DNQX : 2 cells						
			VGAT-Cre	IPSCs amplitude	pharmacological treatment	GABA : 5 cells<br>PTx : 1 cell						
S5	A	VP	VGAT-Cre	5 choice ICSS	Stimulation frequency	7 ChR2 mice						
	B			RTPP	Viral treatment<br>Stimulation parameters	8 YFP mice<br>10 ChR2 mice	**RM 2-way ANOVA**<br>F (1, 16) = 33.59 ; viral treatment<br>F (5, 80) = 2.817 ; stim parameters<br>F (5, 80) = 6.349 ; viral treatment X stim parameters	p < 0.0001<br>p = 0.0215<br>p < 0.0001	***<br>*<br>***	**Sidak:** pre-test vs. test 10mW 20Hz<br>**Sidak:** pre-test vs. test 10mW 40Hz<br>**Sidak:** pre-test vs. test 3mW 40Hz	p = 0.0225<br>p = 0.0002<br>p = 0.0256	*<br>***<br>*
	C			CPP	Viral treatment<br>day	6 YFP mice<br>9 ChR2 mice	**RM 2-way ANOVA**<br>F (1, 13) = 6.173 ; viral treatment X day	p = 0.03	*	**Sidak**: ChR2 pre-test vs. post-test	p = 0.04	*
	D		VGLUT2-Cre	RTPP	Viral treatment<br>Stimulation parameters	8 YFP mice<br>12 ChR2 mice	**RM 2-way ANOVA**<br>F (1, 18) = 13.10 ; viral treatment<br>F (5, 90) = 2.663 ; viral treatment X stim parameters	p = 0.0020<br>p = 0.0272	**<br>*	**Sidak:** pre-test vs. test 10mW 40Hz	p = 0.0153	*
S6	A	LHb	VGAT-Cre	Fos+ cells	Viral treatment / protocol	4 VP-VP YFP passive mice<br>5 VP-VP ChR2 passive mice<br>4 VP-VP ChR2 ICSS mice	**Unpaired *t* test**<br>t (7)=5.248 ; YFP passive vs. ChR2 passive<br>t (6)=7.93 ; YFP passive vs. ChR2 active	p = 0.0012<br>p = 0.0002	**<br>***			
	B				Viral treatment	4 VP-LHb YFP passive mice<br>4 VP-LHb ChR2 passive mice	**Unpaired *t* test**<br>t (6)=2.263 ; YFP passive vs. ChR2 passive	p = 0.06	ns			
	C	VTA			Viral treatment		**Unpaired *t* test**<br>t (6)=1.157 ; YFP passive vs. ChR2 passive	p = 0.29	ns			
	D			Fos+ TH+ cells	Viral treatment		**Unpaired *t* test**<br>t (6)=1.145 ; YFP passive vs. ChR2 passive	p = 0.3	ns			
S7	A-B			schematics								
	C	LHb	VGAT-Cre	Maps & Representative images								
	D	VTA										
	E	VP	wild-type	Representative images and chart pie								
	F	VP, VTA & LHb										

Supplementary Table 3. Related to Figures 6 and 7. Properties of optically evoked EPSCs and IPSCs.

	VP^{VGAT}					
	VTA			LHb		
	GABA			GABA		
	A (pA)	Delay (ms)	τ_d (ms)	A (pA)	Delay (ms)	τ_d (ms)
**n**	19	19	17	13	13	12
**average**	101.97	1.68	14.39	180.84	1.60	11.11
**SEM**	28.75	0.09	1.46	69.35	0.24	1.33

	VP^{VGLUT2}								
	VTA			LHb					
	AMPA			AMPA			GABA		
	A (pA)	Delay (ms)	τ_d (ms)	A (pA)	Delay (ms)	τ_d (ms)	A (pA)	Delay (ms)	τ_d (ms)
**n**	19	19	17	14	14	11	5	5	5
**average**	91.05	1.34	3.54	157.35	1.77	3.44	49.48	1.86	17.17
**SEM**	15.43	0.05	0.53	68.83	0.24	1.04	13.98	0.22	2.79
